# Direct Observation of Vortex Liquid Droplets in the Iron Pnictide Superconductor CaKFe_4_As_4_ at 0.5T_c_

**DOI:** 10.1002/advs.76387

**Published:** 2026-07-03

**Authors:** Óscar Bou Marqués, Jose A. Moreno, Pablo García Talavera, Mingyu Xu, Juan Schmidt, Sergey L. Bud'ko, Paul C. Canfield, Isabel Guillamón, Edwin Herrera, Hermann Suderow

**Affiliations:** ^1^ Laboratorio de Bajas Temperaturas, Departamento de Física de la Materia Condensada, Instituto Nicolás Cabrera and Condensed Matter Physics Center (IFIMAC), Unidad Asociada UAM‐CSIC Universidad Autónoma de Madrid Madrid Spain; ^2^ Ames National Laboratory Iowa State University Ames Iowa USA; ^3^ Department of Physics and Astronomy Iowa State University Ames Iowa USA; ^4^ Departamento de Física, FCEyN Universidad de Buenos Aires Buenos Aires Argentina

**Keywords:** condensed matter physics, dissipation, magnetic field, physics, scanning tunneling microscope, superconductivity, thermal fluctuations, vortex

## Abstract

Type‐II superconductors under magnetic fields remain in a quantum‐coherent, non‐dissipative state as long as vortices are pinned. Dissipation emerges when vortices depin, a process often driven by thermal fluctuations and commonly associated with a melting transition from a vortex solid to a vortex liquid. Macroscopic experiments almost always observe this transition close to the superconducting critical temperature $T_{c}$. However, how the vortex solid responds to thermal fluctuations at the scale of individual vortices, far below the melting transition, remains largely unexplored. Here, we use scanning tunneling microscopy (STM) to directly visualize vortices in the iron‐based superconductor ${\mathrm{CaKFe}}_{4}{\mathrm{As}}_{4}$ ($T_{c}\approx 35\;K$). We observe the formation of vortex liquid droplets—spatially localized regions where vortices exhibit strong thermal fluctuations—at temperatures as low as $0.5\;T_{c}$. These results demonstrate that the onset of dissipation at the local scale occurs at temperatures significantly below $T_{c}$ in type‐II superconductors, revealing a previously unrecognized regime of vortex dynamics.

## Graphical abstract

Vortex matter in the iron‐based superconductor ${\mathrm{CaKFe}}_{4}{\mathrm{As}}_{4}$ is visualized at the nanoscale, revealing the unexpected emergence of vortex‐liquid droplets far below the critical temperature. These localized fluctuating regions coexist with a vortex solid, demonstrating that dissipation‐related dynamics arise well before macroscopic melting and uncovering a previously hidden regime of superconducting behavior.

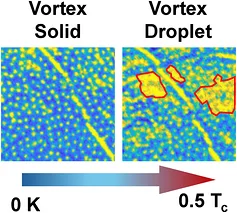

## 1 Introduction

Dissipation in type‐II superconductors is governed by the dynamics of magnetic vortices. Each vortex contains a normal core, where the superconducting order parameter is suppressed over the coherence length ξ, and its mobility sets the fundamental limit to superconducting performance in magnetic fields. In clean systems, vortices form an ordered Abrikosov lattice [1]. Real superconductors, however, are shaped by disorder, which pins vortices, stabilizes the vortex solid and provides a non‐dissipative superconducting state. Thermal fluctuations weaken pinning, modify intervortex interactions, trigger vortex motion, and eventually drive a melting transition into a vortex liquid [2, 3, 4]. In the vortex liquid phase, vortices respond freely to arbitrarily small forces, producing dissipation and destroying the macroscopic superconducting state. Until now, the vortex liquid has been observed very close to the superconducting critical temperature *T* 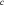 where thermal fluctuations are strongest and superconductivity is weakened [2, 3, 4, 5, 6, 7, 8, 9, 10, 11, 12, 13, 14, 15, 16, 17, 18, 19, 20, 21, 22, 23, 24, 25, 26, 27, 28, 29, 30, 31, 32, 33, 34]. The disorder landscape, however, contains potential troughs with small energy differences [2, 3]. It therefore remains unclear whether vortices are immune to thermal excitations at low temperatures, or whether thermal fluctuations affect their positions well below *T* 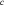. Here, we use temperature‐dependent Scanning Tunneling Microscopy (STM) to reveal vortex liquid droplets (i.e., small regions where vortices exhibit strong fluctuations while the surrounding lattice remains solid) well below *T* 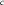 and study their evolution with temperature and magnetic field.

The strength of thermal fluctuations in a superconductor is commonly quantified by the Ginzburg–Levanyuk number $G_{i}$ [35, 36]. This dimensionless parameter compares the thermal energy at the superconducting critical temperature $T_{c}$ to the condensation energy contained in a coherence‐length‐sized volume. For isotropic superconductors, it is given by $G_{i}=\frac{k_{B}T_{c}}{H_{c}^{2}\xi^{3}}$, where $H_{c}$ is the thermodynamic critical field, ξ the coherence length, and $k_{B}$ Boltzmann's constant. A larger $G_{i}$ implies a reduced energy cost for thermally excited order‐parameter fluctuations and, consequently, a stronger thermally induced vortex motion [2, 3, 7, 37, 38]. The value of $G_{i}$ directly governs the range that the vortex‐liquid regime occupies in the magnetic field–temperature phase diagram [39]. In conventional low‐$T_{c}$ superconductors, typical values $G_{i}\approx 10^{-7}$ render thermal fluctuations negligible and the vortex liquid phase is practically absent. In contrast, cuprate superconductors with much larger values, $G_{i}\approx 10^{-3}$–$10^{-2}$, exhibit a larger vortex‐liquid region, which extends, at magnetic fields in the tens of T range, only several percent below $T_{c}$.

The melting of the vortex solid has been investigated using a wide range of experimental techniques. Bulk thermodynamic measurements, such as specific heat and magnetization, have provided early evidence for vortex melting transitions [8, 9, 10, 11, 12]. Microscopic but spatially averaged probes, most notably small‐angle neutron scattering, have revealed the loss of long‐range positional order associated with the transition [13, 14, 15]. Complementary insight has been obtained from direct real‐space visualization techniques that enable imaging of vortex arrangements with nanometer resolution [16, 17, 18, 19, 20, 21, 22, 23, 24, 25, 26, 27, 28, 29, 30]. Some spatially resolved techniques, such as magnetic force microscopy, primarily access the low‐field regime, where intervortex distances are much larger than ξ [40]. In contrast, STM is particularly well suited to imaging individual vortices in the magnetic‐field range relevant for technological applications [31, 32, 41]. In many superconductors, the electronic density of states at the Fermi level is strongly enhanced inside vortex cores compared to the surrounding superconducting background. As a result, maps of the zero‐bias tunneling conductance–which is proportional to the local density of states at the Fermi level–allow simultaneous visualization of large numbers of individual vortices over a broad range of magnetic fields [16, 17, 18, 19, 20, 21, 22, 23, 24, 25, 26, 27, 28, 29]. Despite these advantages, STM studies of vortex melting have been largely confined to superconductors with very small Ginzburg–Levanyuk numbers $G_{i}$. In high‐$T_{c}$ cuprate superconductors, where $G_{i}$ is large, temperature‐dependent STM measurements predominantly reveal vortex solid phases with increasing degrees of thermally induced disorder rather than a clear melting transition [42]. A major challenge specific to cuprates arises from the complex structure of the electronic density of states in and around vortex cores and its strong evolution with temperature, which complicates the identification of vortex melting at the local scale [43, 44, 45, 46].

Owing to their relatively large *T* 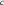 and short coherence length ξ, iron pnictide superconductors exhibit enhanced fluctuations and a vortex liquid range extending a few percent below the *T* 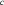  [37, 39]. Among them, the stoichiometric compound ${\mathrm{CaKFe}}_{4}{\mathrm{As}}_{4}$ stands out with a critical temperature of approximately $T_{c}=35$ K, an in‐plane coherence length of $\xi_{ab}\approx 2$ nm and a zero temperature upper critical field exceeding 70 T [47, 48, 49, 50]. Its relatively large Ginzburg–Levanyuk number, $G_{i}\approx 10^{-4}$, reflects substantial thermal fluctuations, while the well‐defined electronic density of states inside vortex cores [51] enables reliable STM imaging. In addition, the dominant pinning centers–intergrown ${\mathrm{CaFe}}_{2}{\mathrm{As}}_{2}$ and ${\mathrm{KFe}}_{2}{\mathrm{As}}_{2}$ layers–are well characterized [52, 53, 54, 55, 56, 57, 58, 59, 60, 61]. These properties establish ${\mathrm{CaKFe}}_{4}{\mathrm{As}}_{4}$ as an ideal model system to investigate thermal vortex fluctuations at the scale of individual vortices. Here, we address the vortex solid and liquid phases of ${\mathrm{CaKFe}}_{4}{\mathrm{As}}_{4}$ using STM and unveil vortex liquid droplets at temperatures well below *T* 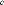.

**FIGURE 1:**
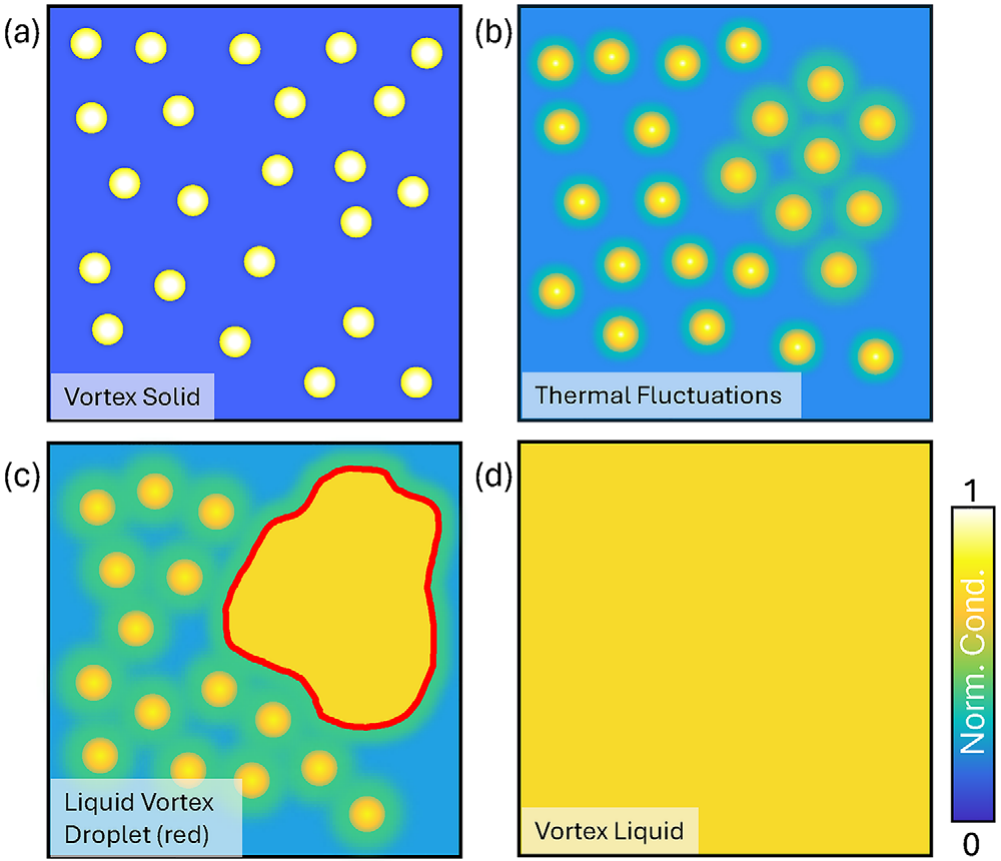
Schematic representation of vortex solid and liquid phases. (a) At low temperatures, the vortices are pinned and eventually form a disordered solid. The superconducting state (dark blue) is clearly distinguished with respect to the normal state at the center of the vortex core (white). (b) As the temperature rises, vortices begin to move, hopping between different positions. Vortex motion leads to fluctuations in the vortex positions, which are much faster than our measurement time, resulting in an apparent increase in the vortex core size (the time‐averaged density of states is represented by the green halo). (c) In certain areas, we eventually observe vortex liquid droplets (large yellow area marked by the red line). These droplets coexist with a vortex solid. (d) Close to the critical temperature, $T_{c}$, of the superconductor, we observe a vortex liquid spanning the whole field of view. In the vortex liquid, the tunneling conductance at zero bias is spatially uniform and lies below the tunneling conductance at bias values well above the superconducting gap, evidencing the presence of a superconducting gap. Color scale provides the zero bias tunneling conductance normalized to its value at high bias, with blue being zero and white being one for the normal phase. Vortex liquid is represented by a yellow color.

STM measurements are inherently time‐averaged, with typical acquisition times of several tens of minutes per conductance map. In the presence of thermal fluctuations, the tunneling conductance measured at a given point represents a time average over a vortex that moves continuously beneath the tip. Locally, the tip typically probes a vortex for only a few seconds, depending on vortex density and image size. Except at locations exhibiting unusually slow dynamics (discussed below), time‐resolved tunneling conductance measurements at fixed positions rarely show appreciable temporal variations, indicating that the dominant fluctuation frequencies lie well above the inverse acquisition time but below the measurement bandwidth, which is of order 10 kHz [62]. When the amplitude of vortex fluctuations is smaller than the vortex core radius, individual cores appear sharp and well defined (Figure 1a). When fluctuations exceed the core size, time averaging leads to an apparent broadening of vortex cores in tunneling conductance maps (schematically shown in Figure 1b and in the vortex‐solid region of Figure 1c). We identify the vortex liquid phase as regions where individual vortices are no longer resolvable at regions where they are clearly observed at lower temperatures. In this regime, illustrated in Figure 1d, the zero‐bias conductance remains suppressed relative to its high‐bias value, represented schematically by the yellow color in Figure 1d, in contrast to the white color of the normal state, consistent with earlier STM observations of vortex liquids [19, 20, 21, 22]. We define vortex liquid droplets as vortex liquid regions exhibiting spatially uniform zero‐bias conductance over length scales exceeding approximately three times the mean intervortex spacing, surrounded by vortex solid regions (Figure 1c).

## 2 Results

**FIGURE 2:**
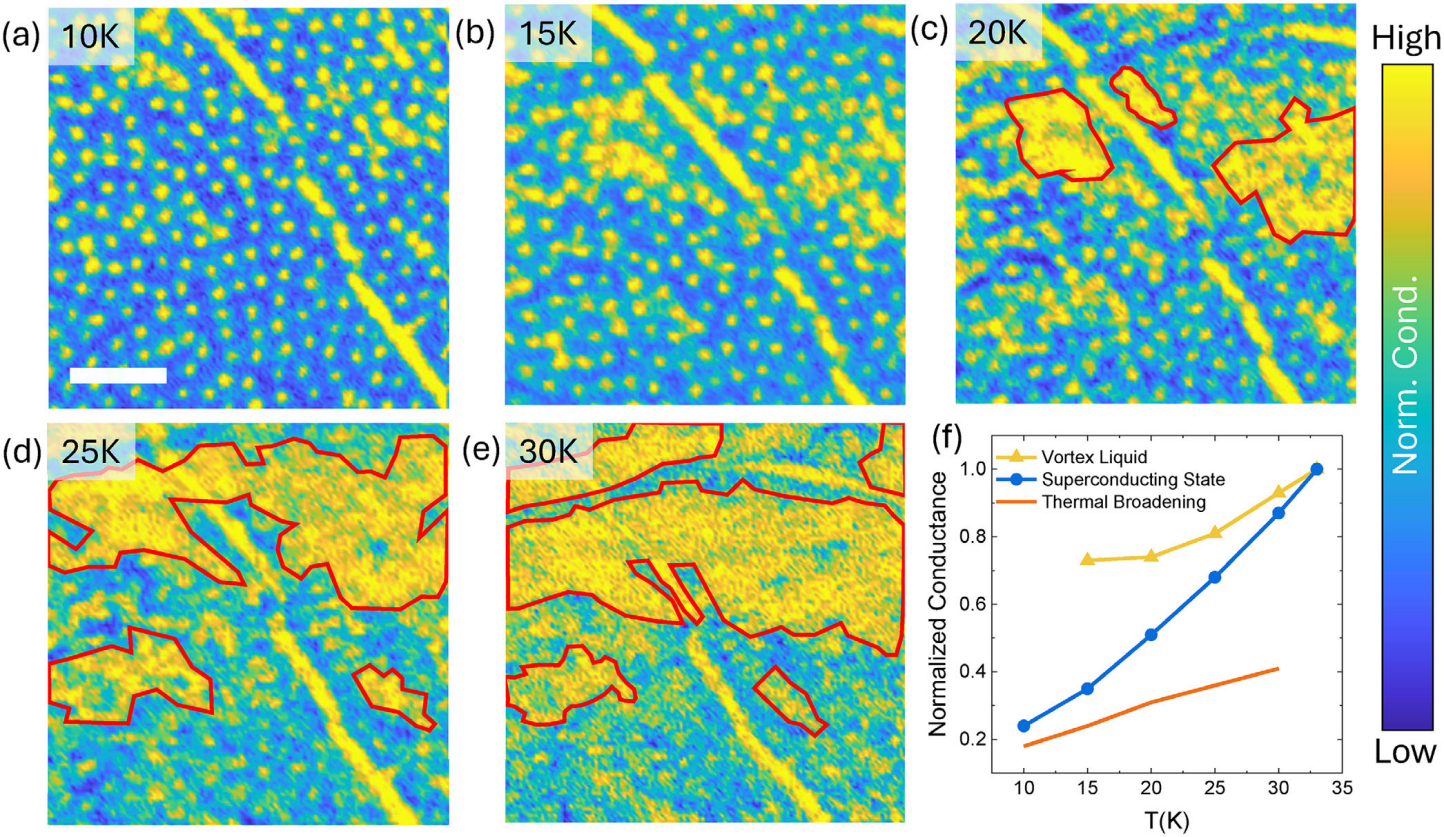
Direct observation of vortex liquid droplets well below $T_{c}$ in ${\mathrm{CaKFe}}_{4}{\mathrm{As}}_{4}$. In (a–e) we show maps of the zero bias tunneling conductance for different temperatures and at 10 T, in approximately the same field of view. Linear defects found in the surface of the sample lead to the roughly diagonal yellow lines shown in the maps (more information in the Section SI and Figures S1 and S2; maps at other temperatures and magnetic fields are commented and provided in the Section SIII and in Figure S4). At higher temperatures, we mark with red contours the areas where we no longer identify isolated vortices in the tunneling conductance maps. In these areas, the tunneling conductance is still well below that of the normal phase. The white scale bar in panel (a) corresponds to 40 nm. The color scale corresponds to slightly different conductance values in each figure, to maximize contrast. To discuss the value of the conductance in (a‐e), we show in (f) the tunneling conductance at zero bias normalized to its high bias value versus temperature, taken at different positions. Lines connect points and are guides to the eye. We show the tunneling conductance taken in between vortices (blue areas in (a–e)) as blue circles. The change induced by the temperature smearing in the zero bias tunneling conductance is shown as an orange line. We also show as yellow triangles the tunneling conductance inside the vortex liquid droplets (average over areas within the red lines in a–e).

Figure 2a–e presents a representative sequence of zero‐bias tunneling conductance maps acquired at different temperatures within the same field of view and at a magnetic field of 10 T. At 10 K (Figure 2a), individual vortices are clearly resolved throughout the entire image. The intervortex spacing is consistent with that expected for a triangular Abrikosov lattice at this magnetic field, in agreement with previous low‐temperature measurements [51]. In addition, the conductance maps reveal linear features with a tunneling conductance slightly below, yet close to, the normal‐state value. These features correlate with line‐like structures observed in the STM topography (see Section SI and Figure S1), and are therefore attributed to underlying structural inhomogeneities. Upon increasing the temperature to 15 K and above (Figure 2b–e), spatially localized regions emerge in which individual vortices progressively lose their identity and merge into extended patches of enhanced conductance. We outline these vortex liquid droplets by red contours. Remarkably, the vortex liquid droplets coexist with a disordered vortex solid surrounding them. Vortex liquid droplets are already observed at temperatures as low as $0.5\;T_{c}$, indicating the onset of strong local vortex fluctuations far below the global superconducting transition. At the highest temperature studied, 30 K–close to the critical temperature $T_{c}\approx 32$ K determined from resistivity measurements at this magnetic field (10 T) [50]–vortex liquid regions expand significantly and encompass nearly half of the field of view. This evolution highlights the progressive growth and merging of vortex liquid droplets with increasing temperature.

To quantify the effect of temperature on the local electronic properties, we plot in Figure 2f the normalized zero‐bias tunneling conductance extracted from different regions of the field of view as a function of temperature. The tunneling conductance σ(V) measured by STM is given by $\sigma \left(V\right)=\int dE\;N\left(E\right)\;\frac{\partial f(E-eV)}{\partial E}$, where N(E) is the quasiparticle density of states, f(E) the Fermi–Dirac distribution, and E the energy. The density of states of ${\mathrm{CaKFe}}_{4}{\mathrm{As}}_{4}$ has been characterized previously and is well described by a modified BCS‐like form [49, 51, 63] (see also Section SII and Figures S3 and S4). In the absence of defects or impurities, the zero‐energy density of states vanishes in the superconducting state [49, 51]. With increasing temperature, the superconducting gap is progressively reduced, and thermal broadening of the quasiparticle spectrum leads to an increase in the zero‐bias tunneling conductance. This effect originates from the temperature evolution of $\frac{\partial f(E-eV)}{\partial E}$, which changes from a near‐δ function at zero temperature to a progressively broadened peak at higher temperatures. The resulting increase in the zero‐bias conductance expected solely from thermal smearing is indicated by the orange line in Figure 2f. This behavior reflects a reduction of the spectroscopic contrast used to image vortices. However, the experimentally observed temperature dependence is strongly position‐dependent. In particular, the zero‐bias tunneling conductance measured in between vortices (blue circles in Figure 2f) increases significantly more rapidly with temperature than expected from thermal smearing alone. This enhanced increase is attributed to the thermally induced overlap of fluctuating vortex cores, which produces excess low‐energy spectral weight even at positions that are vortex‐free at lower temperatures. A detailed discussion of this effect is provided in Section SIII and Figure S5; additional conductance maps acquired at different temperatures and magnetic fields are shown in Section SIV and Figure S6. Within the vortex liquid droplets, the zero‐bias tunneling conductance exhibits a distinct behavior (yellow triangles in Figure 2f). Its magnitude and temperature dependence clearly differ from those measured between vortices, reflecting the rapid motion of vortices within these regions on timescales shorter than the STM acquisition time. Notably, the zero‐bias conductance inside the liquid droplets remains systematically below the normal‐state value, demonstrating that superconducting correlations persist despite the local loss of static vortex order and providing direct spectroscopic evidence for a dynamically fluctuating vortex liquid.

We identify a correlation between the vortex liquid droplets and the underlying pinning landscape. At low temperatures, vortex liquid droplets form in regions that are well separated from the linear defects visible in the tunneling conductance maps (Figure 2b). As the temperature is increased (Figure 2e), these droplets expand and eventually merge with the linear features. We find that vortex liquid droplets preferentially nucleate in regions located between linear defects, particularly in areas where vortices already exhibit enhanced positional fluctuations at temperatures slightly below the onset of droplet formation. This behavior suggests that the linear features act as strong, extended pinning centers that locally stabilize the vortex lattice. As a result, vortices tend to arrange and fluctuate more freely in the regions between these linear defects, where pinning is weaker. Additional, more localized pinning centers in these interstitial regions permit enhanced thermal vortex motion, ultimately enabling the nucleation of vortex liquid droplets.

**FIGURE 3:**
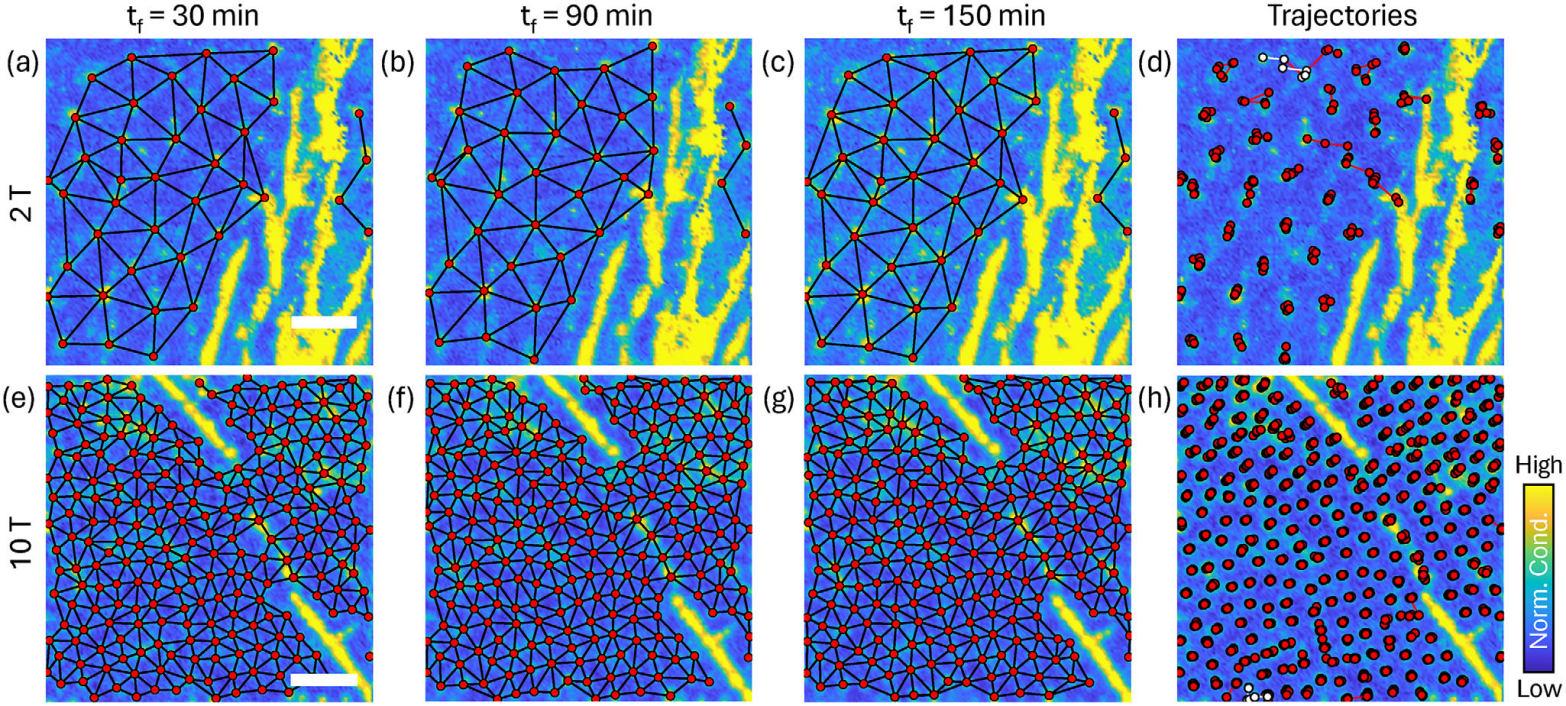
Thermal fluctuations of vortices. In (a–c) and in (e–g) we show zero bias tunneling conductance maps acquired sequentially at, respectively, 2 T and 10 T. Each map has been taken in half an hour, and the field of view remains the same at each magnetic field. Vortex positions are marked by red circles. The black lines join nearest neighbour vortices, obtained by triangulation. In (d,h) we superimpose red circles united by a red line for each magnetic field ((d) for 2 T and (h) for 10 T) on the first zero bias tunneling conductance map of the sequence. These circles and red lines mark the trajectory of each vortex in the sequence. We see that most vortices remain at the same position. However, a few vortices have moved. For example, the vortices marked in white in the top central part of (d), or at the bottom center in (h), are the vortices in each case that present the largest displacement in the field of view. Their travelled distance provides $d_{\max}$ in each case. Note that the largest displacement is much larger at 2 T than at 10 T. In total, we acquired six consecutive images at each magnetic field. White bars in (a) and (e) correspond to 40 nm.

**FIGURE 4:**
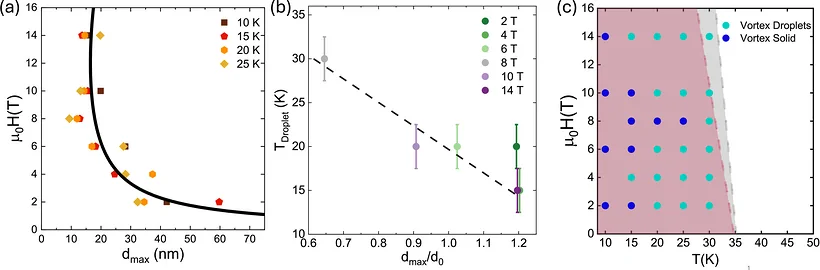
Vortex melting phase diagram obtained from local measurements. In (a) we show the distance traveled by vortices through thermal motion as a function of the magnetic field, $d_{\max}$, for the temperatures provided in the legend. These data are taken inside areas with a well defined vortex solid. The black line corresponds to the displacement calculated using the vortex pinning force density $f_{n}$ given by the Dew‐Hughes model  [64] with parameters $H_{c2}=50$ T, p=0.95 and q=3. In (b), we show the temperature at which we observe the first droplets $T_{Droplet}$ at each of the studied magnetic fields (provided in the legend) as a function of the accumulated distance normalized to the intervortex distance, $d_{\max}/d_{0}$. The black dashed line is a guide to the eye. In (c), we show the phase diagram from macroscopic measurements, obtained from refs.  [50, 65], with grey and maroon areas representing vortex solid and vortex liquid phases. We show as circles the temperatures and fields that we have explored. Dark blue circles signal areas where we observe a vortex solid, resolving individual vortices over nearly the whole field of view. With cyan circles, we show temperatures and fields where we have found vortex liquid droplets or areas with strongly fluctuating vortices.

To further elucidate the role of pinning in the melting process, we now analyze the dynamics of vortices using sequences of zero‐bias tunneling conductance maps acquired consecutively at fixed temperature and magnetic field. Figure 3 displays representative time series obtained at 10 K for magnetic fields of 2 T (Figure 3a–d) and 10 T (Figure 3e–h). The positions of all vortices identified in each frame are marked by red circles. The full set of acquired maps and their analysis are presented in Section SV and Figures S7– S12. Nearest‐neighbor vortex configurations are determined using Delaunay triangulation and are indicated by black lines in Figure 3a–c,e–g. At both magnetic fields, the majority of vortices remain localized near fixed positions over the entire measurement duration. However, at 2 T (Figure 3a–c), several vortices exhibit pronounced motion. As shown in Figure 3d, these vortices travel distances exceeding the average intervortex spacing, signaling substantial thermally activated mobility. To quantify this behavior, we compute for each vortex the accumulated maximal displacement $d_{\max}$ over the duration of the measurement sequence (approximately 150 min), and consider the maximum such value observed at a given temperature and magnetic field. The resulting $d_{\max}$ values are plotted in Figure 4a. We find that vortex mobility decreases systematically with increasing magnetic field. At low fields, thermal fluctuations readily induce large vortex displacements, whereas at fields of 8 T and 10 T the most mobile vortices remain confined to distances smaller than the average intervortex separation (approximately 18 nm at 8 T and 16 nm at 10 T). Notably, data obtained at different temperatures collapse approximately onto a single curve, indicating that the magnetic field plays a dominant role in determining thermally induced vortex motion. In Figure 4b, we relate vortex mobility to the onset of local melting by plotting the temperature at which vortex liquid droplets are first observed as a function of the normalized maximal displacement $d_{\max}/d_{0}$, where $d_{0}$ is the intervortex distance at the corresponding field. A clear correlation emerges: vortex liquid droplets nucleate when thermally activated vortex displacements approach a significant fraction of the intervortex spacing, providing direct evidence that enhanced local vortex mobility precedes and enables the formation of liquid droplets.

We compare our local STM observations with macroscopic measurements in Figure 4c. The pink and grey shaded regions denote the vortex liquid and vortex solid phases inferred from bulk experiments [50, 65], respectively, while the symbols indicate the temperature and magnetic‐field range explored in the present study. Strikingly, the vortex liquid droplets revealed by STM extend down to temperatures as low as $0.5\;T_{c}$, well below the field–temperature region where a vortex liquid phase manifests in macroscopic measurements.

We note that vortex fluctuations do not always occur on time scales shorter than the STM acquisition time. In Section SVI, we discuss an extreme example of unusually slow vortex dynamics, in which an individual vortex is observed to jump intermittently between two distinct positions that evolve over timescales of several hours. This observation highlights the broad distribution of characteristic timescales associated with vortex motion in the presence of pinning and thermal fluctuations.

In the present study, measurements were generally performed upon increasing temperature, and we do not observe any evidence of temperature‐induced hysteresis. While a more systematic investigation of possible hysteretic effects would require additional measurements beyond the scope of this work, their absence is not unexpected given the long acquisition times involved. These extended timescales allow the system to relax toward local equilibrium at each temperature, thereby minimizing the likelihood of history‐dependent effects in our measurements.

## 3 Discussion

The vortex liquid droplets observed here occur at temperatures far below those at which vortex liquid behavior is identified by macroscopic magnetization measurements [48, 50, 65, 66, 67] and by magnetic force microscopy studies of individual vortices at very low magnetic fields (up to 0.008 T) [53]. This discrepancy highlights the intrinsically local nature of vortex melting and demonstrates that dissipation‐related vortex dynamics emerge at the microscopic level long before they become detectable using bulk probes.

Transport and magnetization studies have reported ultrahigh critical current densities in single crystals of ${\mathrm{CaKFe}}_{4}{\mathrm{As}}_{4}$ [50, 57]. Remarkably, this material exhibits a non‐monotonic dependence of the critical current on temperature and magnetic field: at high magnetic fields (≳2 T), the critical current measured at 20 K exceeds that at 5 K [68, 69]. This anomalous behavior is significantly more pronounced than the “fish‐tail” magnetization effects previously observed in cuprates [70] and other iron‐based superconductors [71, 72, 73, 74, 75, 76], and points to a highly unconventional vortex pinning landscape. Consistent with this picture, planar defects arising from ${\mathrm{CaFe}}_{2}{\mathrm{As}}_{2}$ and ${\mathrm{KFe}}_{2}{\mathrm{As}}_{2}$ intergrown layers act as relevant vortex pinning centers, in addition to usual crystalline defects and impurities (see also Section SI and Figure S2) [52, 53, 54, 55, 56, 57, 58, 59, 68, 69, 77]. Such an inhomogeneous pinning landscape has a direct impact on vortex dynamics and plays a central role in the melting behavior of the vortex solid in ${\mathrm{CaKFe}}_{4}{\mathrm{As}}_{4}$.

The maximal thermally induced vortex displacement $d_{\max}$ (Figure 4a) exhibits a pronounced magnetic‐field dependence that is nearly independent of temperature. This behavior closely resembles the field dependence predicted by the Dew‐Hughes model of vortex pinning [64], in which the competition between elastic intervortex interactions and the pinning potential leads to a universal scaling of the pinning force as $\propto h^{p}{\left(1-h\right)}^{q}$, with $h=H/H_{c2}$ and p and q characteristic exponents. Our data follow this scaling, as shown by the black curve in Figure 4a, using $H_{c2}=50$ T, p=0.95, and q=3. Assuming that $d_{\max}$ inversely reflects the effective pinning force, we find that the field at which pinning is strongest (corresponding to minimal $d_{\max}$) occurs at $h_{\max}\approx 0.24$. This value is close to $h_{\max}\approx 0.28$ reported previously from macroscopic measurements on single crystals [50, 78]. Within the Dew‐Hughes framework, values of $h_{\max}\approx 0.2$ indicate surface‐like pinning, while $h_{\max}\approx 0.33$ corresponds to point‐like pinning [64]. This suggests that the extended ${\mathrm{CaFe}}_{2}{\mathrm{As}}_{2}$/${\mathrm{KFe}}_{2}{\mathrm{As}}_{2}$ intergrowth layers act as surface‐like pinning centers, while point‐like disorder dominates in the regions between them. Although the overall scaling of $d_{\max}$ is robust, the incomplete collapse of the data indicates that multiple pinning mechanisms coexist and jointly influence vortex dynamics.

We also find that a small $d_{\max}$ leads to an increased temperature at which vortex liquid droplets appear $T_{Droplet}$ (Figure 4b). Although we cannot completely rule out effects arising from random distributions of pinning centers within a given field of view, owing to the relatively small number of images we can take, the observed trend is consistent throughout our data.

Altogether, the behavior roughly following Dew‐Hughes scaling and the possible relationship between $T_{Droplet}$ and $d_{\max}$ illustrates how the varied pinning potential landscape governs the motion of vortices and the formation of vortex liquid droplets alike.

Beyond pinning effects, the symmetry of the electronic structure may further influence vortex behavior. In superconductors with strong fourfold anisotropy in the normal‐state density of states, such as ${\mathrm{CaKFe}}_{4}{\mathrm{As}}_{4}$, a transition between hexagonal and square vortex lattices has been predicted and observed at sufficiently high magnetic fields [79, 80, 81]. In the present samples, however, vortex disorder appears too strong to stabilize such a transition over macroscopic length scales [51]. Nevertheless, electronic anisotropies can strongly renormalize vortex elastic moduli; in particular, the rotational modulus may vanish under certain conditions [82], which could locally favor enhanced vortex fluctuations.

In contrast to our observations, melting transitions in effectively 2D vortex systems–studied in thin films under perpendicular magnetic fields–typically manifest through the proliferation of topological defects within an otherwise ordered hexagonal lattice [19, 20, 21, 24, 25, 26], eventually leading to large‐scale vortex liquid regions. The observation of nanoscale vortex liquid droplets in ${\mathrm{CaKFe}}_{4}{\mathrm{As}}_{4}$ instead reveals a distinct melting phenomenology governed by a highly heterogeneous pinning landscape arising from the interplay of crystalline order and extended defects.

Our results raise important questions regarding the emergence of finite voltage in macroscopic critical‐current measurements. Even in the presence of a substantial density of vortex liquid droplets, macroscopic samples may sustain zero resistance due to the formation of percolating superconducting current paths [83].

Nevertheless, small micron‐sized samples, as those used in devices, should be much more sensitive to the formation of vortex liquid droplets [84, 85, 86, 87]. Vortex liquid droplets forming, for example, near junctions can strongly affect the Josephson current. Furthermore, quantum coherent manipulation of vortex states, recently proposed in ref.  [88] using nanostructures at very low magnetic fields, requires strong trapping and absence of thermal fluctuations. Our results suggest that engineering well‐defined pinning landscapes—ideally dominated by a single pinning mechanism–is essential to suppress the formation of vortex liquid droplets well below $T_{c}$.

## 4 Conclusion

In summary, we have visualized superconducting vortices as a function of temperature and magnetic field in the iron‐based superconductor ${\mathrm{CaKFe}}_{4}{\mathrm{As}}_{4}$. At low temperatures, we observe a disordered but pinned vortex solid that undergoes local melting upon heating, giving rise to spatially confined vortex‐liquid droplets at temperatures well below the macroscopic vortex melting transition. Our results demonstrate that vortex melting is inherently non‐uniform and strongly governed by the local pinning landscape. By directly characterizing thermally activated vortex motion, we establish a close connection between vortex pinning, thermal fluctuations, and the emergence of liquid regions. The resulting phase diagram reveals an extended crossover regime in which vortex solid and vortex liquid phases coexist locally. These findings indicate that the truly dissipationless superconducting state persists over a substantially narrower temperature range than suggested by bulk measurements alone, underscoring the importance of engineering robust pinning landscapes for superconducting devices.

## 5 Methods

Single crystals of ${\mathrm{CaKFe}}_{4}{\mathrm{As}}_{4}$ were grown and characterized following the procedures described in refs.  [48, 89]. Samples were cleaved in situ by gluing a post to the crystal surface and mechanically detaching it using the slider mechanism detailed in ref.  [90]. This procedure reliably produced large (>2μm×2μm), clean, and atomically flat surfaces. STM tips were fabricated from gold and conditioned by cleaning and sharpening on a gold surface, following established protocols [91].

All measurements were performed using a modified laboratory‐built STM system, similar to that described in ref.  [90]. The control software is detailed in ref.  [92] and is publicly available [93]. Image rendering was primarily carried out using the same laboratory‐developed software [92], with additional image processing performed when needed using standard STM visualization tools [94].

The STM was operated inside a liquid‐helium bath cryostat at 4.2 K. The microscope was mounted on a thermally stabilized support anchored to a cold plate, itself thermally coupled to the helium bath [95]. Temperature control was achieved using a resistive heater in conjunction with a temperature controller. To minimize thermal creep arising from temperature gradients, the heater was positioned between the cold source and the STM sample holder. Temperature stability better than ±100 mK was maintained up to temperatures exceeding 40 K. Thermal stability was verified by acquiring consecutive topographic images and confirming that the field of view remained unchanged. When necessary, the field of view was adjusted using reference features in the topography.

Vortices were imaged by acquiring spatially resolved tunneling conductance spectra as a function of bias voltage. Conductance maps were generated by normalizing the measured tunneling conductance to a reference value taken at a bias well above the superconducting gap (typically >10 mV) and plotting the resulting zero‐bias conductance as a function of position. Because the tunneling conductance varies strongly with temperature, the color scale was optimized independently for each temperature (unless stated otherwise) to maximize vortex contrast. During this work we show superconducting regions as blue, while vortex cores (locally normal regions) are rendered in white, unless stated otherwise.

For each applied magnetic field, the temperature was increased in steps of 5 K between 10 and 30 K. At every temperature, multiple conductance maps were acquired sequentially to track the positions of individual vortices over time. Each map was typically acquired within 30 min. Vortex positions were analyzed using Delaunay triangulation, implemented with software described in refs.  [20, 96, 97, 98] and available through ref.  [93]. Using the in situ positioning method described in ref.  [90], we investigated multiple fields of view with varying defect densities across two different samples.

The Supplementary Information provides additional data and discussion, and includes refs.  [99, 100, 101, 102, 103, 104, 105, 106, 107, 108, 109].

## Author Contributions

O.B.M. and J.A.M. performed the experiments, with the supervision of E.H., I.G., and H.S. The analysis of the data and the coding of the tools used to do so were made by O.B.M. and P.G.T. Samples were synthesized and characterized by M.X., J.S., S.L.B., and P.C.C. The paper was written by O.B.M., J.A.M., I.G., E.H., and H.S. with input and approval from all authors.

## Conflicts of Interest

The authors declare no conflicts of interest.

## Supporting information


**Supporting File**: advs76387‐sup‐0001‐SuppMat.pdf.

## Data Availability

The data shown in this study is available from the corresponding author upon reasonable request, and can be downloaded from https://doi.org/10.17605/OSF.IO/WZHCJ.

## References

[1] A. Abrikosov , “On the Magnetic Properties of Superconductors of the Second Group,” Soviet Physics—Journal of Experimental and Theoretical Physics 32 (1957): 1442.

[2] G. Blatter , M. V. Feigel'man , V. B. Geshkenbein , A. I. Larkin , and V. M. Vinokur , “Vortices in High‐Temperature Superconductors,” Reviews of Modern Physics 66, no. 4 (1994): 1125.

[3] E. H. Brandt , “The Flux‐Line Lattice in Superconductors,” Reports on Progress in Physics 58, no. 11 (1995): 1465.

[4] D. R. Nelson and V. Vinokur , “Boson Localization and Correlated Pinning of Superconducting Vortex Arrays,” Physical Review B 48, no. 17 (1993): 13060. 10.1103/physrevb.48.13060 10007683

[5] T. Klein , I. Joumard , S. Blanchard , et al., “A Bragg Glass Phase in the Vortex Lattice of a Type II Superconductor,” Nature 413, no. 6854 (2001): 404–406. 11574883 10.1038/35096534

[6] G. P. Mikitik and E. H. Brandt , “Effect of Pinning on the Vortex‐Lattice Melting Line in Type‐II Superconductors,” Physical Review B 68 (2003): 054509.

[7] J. Kierfeld and V. Vinokur , “Lindemann Criterion and Vortex Lattice Phase Transitions in Type‐II Superconductors,” Physical Review B 69 (2004): 024501.

[8] E. Zeldov , D. Majer , M. Konczykowski , et al., “Thermodynamic Observation of First‐Order Vortex‐Lattice Melting Transition in ${\mathrm{Bi}}_{2}{\mathrm{Sr}}_{2}{\mathrm{CaCu}}_{2}O_{8}$ ,” Nature 375, no. 6530 (1995): 373–376.

[9] H. Safar , P. Gammel , D. Huse , et al., “Experimental Evidence for a First‐Order Vortex‐Lattice‐Melting Transition in Untwinned, Single Crystal ${\mathrm{YBa}}_{2}{\mathrm{Cu}}_{3}O_{7}$ ,” Physical Review Letters 69, no. 5 (1992): 824. 10047042 10.1103/PhysRevLett.69.824

[10] M. Willemin , A. Schilling , H. Keller , et al., “First‐Order Vortex‐Lattice Melting Transition in ${\mathrm{YBa}}_{2}{\mathrm{Cu}}_{3}O_{7-\delta }$ Near the Critical Temperature Detected by Magnetic Torque,” Physical Review Letters 81, no. 19 (1998): 4236.

[11] Y. Paltiel , E. Zeldov , Y. N. Myasoedov , et al., “Dynamic Instabilities and Memory Effects in Vortex Matter,” Nature 403, no. 6768 (2000): 398–401. 10667785 10.1038/35000145

[12] A. Schilling , R. A. Fisher , N. E. Phillips , et al., “Anisotropic Latent Heat of Vortex‐Lattice Melting in Untwinned ${\mathrm{YBa}}_{2}{\mathrm{Cu}}_{3}O_{7-\delta }$ ,” Physical Review Letters 78 (1997): 4833–4836.

[13] R. Cubitt , E. M. Forgan , G. Yang , et al., “Direct Observation of Magnetic Flux Lattice Melting and Decomposition in the High‐$T_{c}$ Superconductor ${\mathrm{Bi}}_{2.15}{\mathrm{Sr}}_{1.95}{\mathrm{CaCu}}_{2}O_{8+x}$ ,” Nature 365, no. 6445 (1993): 407–411.

[14] N. Avraham , B. Khaykovich , Y. Myasoedov , et al., ““Inverse” Melting of a Vortex Lattice,” Nature 411, no. 6836 (2001): 451–454. 11373671 10.1038/35078021

[15] U. Yaron , P. L. Gammel , D. A. Huse , et al., “Structural Evidence for a Two‐Step Process in the Depinning of the Superconducting Flux‐Line Lattice,” Nature 376, no. 6543 (1995): 753–755.

[16] H. F. Hess , R. B. Robinson , R. C. Dynes , J. M. Valles , and J. V. Waszczak , “Scanning‐Tunneling‐Microscope Observation of the Abrikosov Flux Lattice and the Density of States Near and Inside a Fluxoid,” Physical Review Letters 62 (1989): 214–216. 10039952 10.1103/PhysRevLett.62.214

[17] O. Fischer , M. Kugler , I. Maggio‐Aprile , C. Berthod , and C. Renner , “Scanning Tunneling Spectroscopy of High‐Temperature Superconductors,” Reviews of Modern Physics 79 (2007): 353–419.

[18] H. Suderow , I. Guillamón , J. G. Rodrigo , and S. Vieira , “Imaging Superconducting Vortex Cores and Lattices with a Scanning Tunneling Microscope,” Superconductor Science and Technology 27, no. 6 (2014): 063001.

[19] I. Guillamón , H. Suderow , A. Fernández‐Pacheco , et al., “Direct Observation of Melting in a Two‐Dimensional Superconducting Vortex Lattice,” Nature Physics 5, no. 9 (2009): 651–655.

[20] I. Guillamón , R. Córdoba , J. Sesé , et al., “Enhancement of Long‐Range Correlations in a 2D Vortex Lattice by an Incommensurate 1D Disorder Potential,” Nature Physics 10, no. 11 (2014): 851–856.

[21] R. Duhan , S. Sengupta , J. Jesudasan , S. Basistha , and P. Raychaudhuri , “Inverse Melting and Re‐Entrant Transformations of the Vortex Lattice in Amorphous Re6Zr Thin Film,” Nature Communications 16, no. 1 (2025): 2100. 10.1038/s41467-025-57431-3 PMC11873147 40025129

[22] I. Roy , S. Dutta , A. N. Roy Choudhury , et al., “Melting of the Vortex Lattice Through Intermediate Hexatic Fluid in an a−MoGe Thin Film,” Physical Review Letters 122 (2019): 047001. 30768342 10.1103/PhysRevLett.122.047001

[23] I. Guillamón , H. Suderow , S. Vieira , et al., “Direct Observation of Stress Accumulation and Relaxation in Small Bundles of Superconducting Vortices in Tungsten Thin Films,” Physical Review Letters 106 (2011): 077001. 21405532 10.1103/PhysRevLett.106.077001

[24] A. Yazdani , W. R. White , M. R. Hahn , et al., “Observation of Kosterlitz‐Thouless‐Type Melting of the Disordered Vortex Lattice in Thin Films of a‐MoGe,” Physical Review Letters 70 (1993): 505–508. 10054129 10.1103/PhysRevLett.70.505

[25] P. Berghuis , A. L. F. van der Slot , and P. H. Kes , “Dislocation‐Mediated Vortex‐Lattice Melting in Thin Films of a‐${\mathrm{Nb}}_{3}\mathrm{Ge}$ ,” Physical Review Letters 65 (1990): 2583–2586. 10042634 10.1103/PhysRevLett.65.2583

[26] C. Dasgupta and O. T. Valls , “Two‐Step Melting of the Vortex Solid in Layered Superconductors with Random Columnar Pins,” Physical Review Letters 91 (2003): 127002. 14525390 10.1103/PhysRevLett.91.127002

[27] M. Marchevsky , A. Keurentjes , J. Aarts , and P. H. Kes , “Elastic Deformations in Field‐Cooled Vortex Lattices in ${\mathrm{NbSe}}_{2}$ ,” Physical Review B 57 (1998): 6061–6066.

[28] J. Hecher , M. Zehetmayer , and H. W. Weber , “How the Macroscopic Current Correlates with the Microscopic Flux‐Line Distribution in a Type‐II Superconductor: An Experimental Study,” Superconductor Science and Technology 27, no. 7 (2014): 075004.

[29] M. Zehetmayer , “How the Vortex Lattice of a Superconductor Becomes Disordered: A Study by Scanning Tunneling Spectroscopy,” Scientific Reports 5, no. 1 (2015): 9244. 25784605 10.1038/srep09244 PMC5378196

[30] A. P. Petrović , Y. Fasano , R. Lortz , et al., “Real‐Space Vortex Glass Imaging and the Vortex Phase Diagram of ${\mathrm{SnMo}}_{6}S_{8}$ ,” Physical Review Letters 103 (2009): 257001. 20366275 10.1103/PhysRevLett.103.257001

[31] D. Dew‐Hughes , “The Critical Current of Superconductors: An Historical Review,” Low Temperature Physics 27, no. 9 (2001): 713–722.

[32] A. Campbell and J. Evetts , “Flux Vortices and Transport Currents in Type‐II Superconductors,” Advances in Physics 21, no. 90 (1972): 199–428.

[33] O. B. Hyun , D. K. Finnemore , L. Schwartzkopf , and J. R. Clem , “Elementary Pinning Force for a Superconducting Vortex,” Physical Review Letters 58 (1987): 599–601. 10034982 10.1103/PhysRevLett.58.599

[34] M. Breitwisch and D. K. Finnemore , “Pinning of a Single Abrikosov Vortex in Superconducting Nb Thin Films Using Artificially Induced Pinning Sites,” Physical Review B 62 (2000): 671–677.

[35] V. Ginzburg , “Some Remarks on Phase Transitions of the Second Kind and the Microscopic Theory of Ferroelectric Materials,” Soviet Physics Solid State 2 (1961): 1824–1834.

[36] A. Levanyuk , “Contribution to the Theory of Light Scattering Near the Second‐Order Phase‐Transition Points,” Soviet Physics–JETP 9, no. 3 (1959): 571–576.

[37] S. Eley , M. Miura , B. Maiorov , and L. Civale , “Universal Lower Limit on Vortex Creep in Superconductors,” Nature Materials 16, no. 4 (2017): 409–413. 28191897 10.1038/nmat4840

[38] G. P. Mikitik and E. H. Brandt , “Peak Effect, Vortex‐Lattice Melting Line, and Order‐Disorder Transition in Conventional and High‐$T_{c}$ Superconductors,” Physical Review B 64 (2001): 184514.

[39] A. Koshelev , K. Willa , R. Willa , et al., “Melting of the Vortex Lattice in the Magnetic Superconductor ${\mathrm{RbEuFe}}_{4}{\mathrm{As}}_{4}$ ,” Physical Review B 100, no. 9 (2019): 094518.

[40] L. Embon , Y. Anahory , Ž. L. Jelić , et al., “Imaging of Super‐Fast Dynamics and Flow Instabilities of Superconducting Vortices,” Nature Communications 8, no. 1 (2017): 85. 10.1038/s41467-017-00089-3 PMC5519736 28729642

[41] L. Bossoni , P. Carretta , and M. Poggio , “Vortex Lattice Melting of a ${\mathrm{NbSe}}_{2}$ Single Grain Probed by Ultrasensitive Cantilever Magnetometry,” Applied Physics Letters 104, no. 18 (2014): 182601.

[42] K. Shibata , T. Nishizaki , M. Maki , and N. Kobayashi , “Vortex Matter in ${\mathrm{YBa}}_{2}{\mathrm{Cu}}_{3}O_{Y}$ Single Crystals Investigated by Scanning Tunneling Spectroscopy,” Physical Review B 72 (2005): 014525.

[43] S. H. Pan , E. W. Hudson , A. K. Gupta , et al., “STM Studies of the Electronic Structure of Vortex Cores in ${\mathrm{Bi}}_{2}{\mathrm{Sr}}_{2}{\mathrm{CaCu}}_{2}O_{8+\delta }$ ,” Physical Review Letters 85 (2000): 1536–1539. 10970548 10.1103/PhysRevLett.85.1536

[44] S. Yoshizawa , T. Koseki , K. Matsuba , et al., “High‐Resolution Scanning Tunneling Spectroscopy of Vortex Cores in Inhomogeneous Electronic States of ${\mathrm{Bi}}_{2}{\mathrm{Sr}}_{2}{\mathrm{CaCu}}_{2}O_{x}$ ,” Journal of the Physical Society of Japan 82, no. 8 (2013): 083706.

[45] S. D. Edkins , A. Kostin , K. Fujita , et al., “Magnetic Field–Induced Pair Density Wave State in the Cuprate Vortex Halo,” Science 364, no. 6444 (2019): 976–980. 31171694 10.1126/science.aat1773

[46] I. Maggio‐Aprile , C. Renner , A. Erb , E. Walker , and O. Fischer , “Direct Vortex Lattice Imaging and Tunneling Spectroscopy of Flux Lines on ${\mathrm{YBa}}_{2}{\mathrm{Cu}}_{3}O_{7}$ ,” Physical Review Letters 75 (1995): 2754–2757. 10059396 10.1103/PhysRevLett.75.2754

[47] A. Iyo , K. Kawashima , T. Kinjo , et al., “New‐Structure‐Type Fe‐Based Superconductors: ${\mathrm{CaAFe}}_{4}{\mathrm{As}}_{4}$ (A=K, Rb, Cs) and ${\mathrm{SrAFe}}_{4}{\mathrm{As}}_{4}$ (A=Rb, Cs),” Journal of the American Chemical Society 138, no. 10 (2016): 3410–3415. 26943024 10.1021/jacs.5b12571

[48] W. R. Meier , T. Kong , U. S. Kaluarachchi , et al., “Anisotropic Thermodynamic and Transport Properties of Single‐Crystalline ${\mathrm{CaKFe}}_{4}{\mathrm{As}}_{4}$ ,” Physical Review B 94, no. 6 (2016): 064501.

[49] K. Cho , A. Fente , S. Teknowijoyo , et al., “Nodeless Multiband Superconductivity in Stoichiometric Single‐Crystalline ${\mathrm{CaKFe}}_{4}{\mathrm{As}}_{4}$ ,” Physical Review B 95 (2017): 100502.

[50] S. J. Singh , M. Bristow , W. R. Meier , et al., “Ultrahigh Critical Current Densities, the Vortex Phase Diagram, and the Effect of Granularity of the Stoichiometric High‐$T_{c}$ Superconductor ${\mathrm{CaKFe}}_{4}{\mathrm{As}}_{4}$ ,” Physical Review Materials 2 (2018): 074802.

[51] A. Fente , W. R. Meier , T. Kong , et al., “Influence of Multiband Sign‐Changing Superconductivity on Vortex Cores and Vortex Pinning in Stoichiometric High‐$T_{c}$ ${\mathrm{CaKFe}}_{4}{\mathrm{As}}_{4}$ ,” Physical Review B 97 (2018): 134501.

[52] A. Ichinose , S. Pyon , T. Tamegai , and S. Ishida , “Elucidating the Origin of Planar Defects That Enhance Critical Current Density in ${\mathrm{CaKFe}}_{4}{\mathrm{As}}_{4}$ Single Crystals,” Superconductor Science and Technology 34, no. 3 (2021): 034003.

[53] T. He , X.‐S. Gao , K.‐H. Yin , et al., “Direct Observation of Low‐Field Vortex Patterns in Stoichiometric ${\mathrm{CaKFe}}_{4}{\mathrm{As}}_{4}$ Single Crystal,” Physical Review B 111 (2025): 104512.

[54] T. He , T. Chen , X.‐J. Liu , et al., “Defect‐Induced Vortex Pattern Variations in a ${\mathrm{CaKFe}}_{4}{\mathrm{As}}_{4}$ Single Crystal,” Physical Review B 112, no. 17 (2025): 174516.

[55] A. Takahashi , S. Pyon , Y. Kobayashi , et al., “Effects of Splayed Columnar Defects on Critical Current Density in ${\mathrm{CaKFe}}_{4}{\mathrm{As}}_{4}$ ,” Journal of Physics: Conference Series 1590, no. 1 (2020): 012015.

[56] Y. Kobayashi , S. Pyon , A. Takahashi , and T. Tamegai , “Effects of Point Defects Introduced by Co‐Doping and Proton Irradiation in ${\mathrm{CaKFe}}_{4}{\mathrm{As}}_{4}$ ,” Journal of Physics: Conference Series 1590, no. 1 (2020): 012014.

[57] N. Haberkorn , M. Xu , W. R. Meier , et al., “Enhancement of Critical Current Density in ${\mathrm{CaKFe}}_{4}{\mathrm{As}}_{4}$ Single Crystals Through 3 MeV Proton Irradiation,” Superconductor Science and Technology 33, no. 2 (2020): 025008.

[58] N. Haberkorn , M. Xu , W. R. Meier , et al., “Effect of Ni Doping on Vortex Pinning in CaK( Single Crystals,” Physical Review B 100 (2019): 064524.

[59] Y. Chen , C. Wang , Y. Zu , et al., “Evolution of Critical Current Density in ${\mathrm{CaKFe}}_{4}{\mathrm{As}}_{4}$ with La‐Doping,” Superconductor Science and Technology 38, no. 1 (2024): 015004.

[60] S. Huyan , N. Haberkorn , M. Xu , P. C. Canfield , and S. L. Bud'ko , “Effects of Pressure on Superconducting Properties and Vortex Pinning of ${\mathrm{CaKFe}}_{4}{\mathrm{As}}_{4}$ Single Crystals,” Journal of the Physical Society of Japan 94, no. 2 (2025): 024708.

[61] J. Cui , Q.‐P. Ding , W. R. Meier , et al., “Magnetic Fluctuations and Superconducting Properties of ${\mathrm{CaKFe}}_{4}{\mathrm{As}}_{4}$ Studied by As 75 NMR,” Physical Review B 96, no. 10 (2017): 104512.

[62] A. Kohen , T. Cren , T. Proslier , et al., “Superconducting Vortex Profile from Fixed Point Measurements: The “Lazy Fisherman” Tunneling Microscopy Method,” Applied Physics Letters 86, no. 21 (2005): 212503.

[63] D. Mou , T. Kong , W. R. Meier , et al., “Enhancement of the Superconducting Gap by Nesting in ${\mathrm{CaKFe}}_{4}{\mathrm{As}}_{4}:$ A New High‐Temperature Superconductor,” Physical Review Letters 117 (2016): 277001. 28084772 10.1103/PhysRevLett.117.277001

[64] D. Dew‐Hughes , “Flux Pinning Mechanisms in Type‐II Superconductors,” Philosophical Magazine 30, no. 2 (1974): 293–305.

[65] W. Cheng , H. Lin , B. Shen , and H.‐H. Wen , “Comparative Study of Vortex Dynamics in ${\mathrm{CaKFe}}_{4}{\mathrm{As}}_{4}$ and ${\mathrm{Ba}}_{0.6}K_{0.4}{\mathrm{Fe}}_{2}{\mathrm{As}}_{2}$ Single Crystals,” Science Bulletin 64, no. 2 (2019): 81–90. 36659641 10.1016/j.scib.2018.12.024

[66] C. Wang , T. He , Q. Han , et al., “Flux Pinning and the Vortex Phase Diagram in Optimized ${\mathrm{CaKFe}}_{4}{\mathrm{As}}_{4}$ Single Crystals Fabricated by a One‐Step Method,” Superconductor Science and Technology 33, no. 4 (2020): 045011.

[67] C. Wang , T. He , Q. Han , et al., “Vortex Creep Activation Energies and Depinning Currents in ${\mathrm{CaKFe}}_{4}{\mathrm{As}}_{4}$ and ${\mathrm{Ba}}_{0.6}K_{0.4}{\mathrm{Fe}}_{2}{\mathrm{As}}_{2}$ Revealed by AC Susceptibility Measurements,” Journal of Physics: Condensed Matter 32, no. 41 (2020): 415607. 10.1088/1361-648X/ab9f50 32575090

[68] S. Ishida , A. Iyo , H. Ogino , et al., “Unique Defect Structure and Advantageous Vortex Pinning Properties in Superconducting ${\mathrm{CaKFe}}_{4}{\mathrm{As}}_{4}$ ,” npj Quantum Materials 4, no. 1 (2019): 27.

[69] S. Pyon , A. Takahashi , I. Veshchunov , et al., “Large and Significantly Anisotropic Critical Current Density Induced by Planar Defects in ${\mathrm{CaKFe}}_{4}{\mathrm{As}}_{4}$ Single Crystals,” Physical Review B 99 (2019): 104506.

[70] M. Daeumling , J. M. Seuntjens , and D. C. Larbalestier , “Oxygen‐Defect Flux Pinning, Anomalous Magnetization and Intra‐Grain Granularity in ${\mathrm{YBa}}_{2}{\mathrm{Cu}}_{3}O_{7-\delta }$ ,” Nature 346, no. 6282 (1990): 332–335.

[71] H. Yang , H. Luo , Z. Wang , and H.‐H. Wen , “Fishtail Effect and the Vortex Phase Diagram of Single Crystal ${\mathrm{Ba}}_{0.6}K_{0.4}{\mathrm{Fe}}_{2}{\mathrm{As}}_{2}$ ,” Applied Physics Letters 93, no. 14 (2008): 142506.

[72] T. Taen , Y. Tsuchiya , Y. Nakajima , and T. Tamegai , “Superconductivity at $T_{c}∼14\;K$ in Single‐Crystalline ${\mathrm{FeTe}}_{0.61}{\mathrm{Se}}_{0.39}$ ,” Physical Review B 80 (2009): 092502.

[73] R. Prozorov , N. Ni , M. A. Tanatar , et al., “Vortex Phase Diagram of $\text{Ba}{\left({\text{Fe}}_{0.93}{\text{Co}}_{0.07}\right)}_{2}{As}_{2}$ Single Crystals,” Physical Review B 78 (2008): 224506.

[74] R. Prozorov , M. A. Tanatar , B. Roy , et al., “Magneto‐Optical Study of $\text{Ba}{\left({\text{Fe}}_{1-x}M_{x}\right)}_{2}{\text{As}}_{2}$ (M=Co and Ni) Single Crystals Irradiated with Heavy Ions,” Physical Review B 81 (2010): 094509.

[75] P. Prommapan , M. A. Tanatar , B. Lee , et al., “Magnetic‐Field‐Dependent Pinning Potential in LiFeAs Superconductor from Its Campbell Penetration Depth,” Physical Review B 84 (2011): 060509.

[76] R. Prozorov , M. Tanatar , E. Blomberg , et al., “Doping‐Dependent Irreversible Magnetic Properties of Ba( Single Crystals,” Physica C: Superconductivity 469, no. 9 (2009): 667–673.

[77] P. K. N. Sugali , S. Ishida , K. Kimoto , et al., “Intrinsic Defect Structures of Polycrystalline ${\mathrm{CaKFe}}_{4}{\mathrm{As}}_{4}$ Superconductors,” Physical Chemistry Chemical Physics 23 (2021): 19827–19833. 34525149 10.1039/d1cp02613e

[78] N. Haberkorn , S. Suárez , S. L. Bud'ko , and P. C. Canfield , “Strong Pinning and Slow Flux Creep Relaxation in Co‐Doped ${\mathrm{CaFe}}_{2}{\mathrm{As}}_{2}$ Single Crystals,” Solid State Communications 318 (2020): 113963.

[79] M. R. Eskildsen , P. L. Gammel , B. P. Barber , et al., “Structural Stability of the Square Flux Line Lattice in ${\mathrm{YNi}}_{2}B_{2}C$ and ${\mathrm{LuNi}}_{2}B_{2}C$ Studied with Small‐Angle Neutron Scattering,” Physical Review Letters 79 (1997): 487–490.

[80] J. M. Densmore , P. Das , K. Rovira , et al., “Small‐Angle Neutron Scattering Study of the Vortex Lattice in Superconducting ${\text{LuNi}}_{2}B_{2}C$ ,” Physical Review B 79 (2009): 174522.

[81] U. Yaron , P. Gammel , A. Ramirez , et al., “Microscopic Coexistence of Magnetism and Superconductivity in ${\mathrm{ErNi}}_{2}B_{2}C$ ,” Nature 382, no. 6588 (1996): 236–238.

[82] P. Miranović and V. G. Kogan , “Elastic Moduli of Vortex Lattices Within Nonlocal London Model,” Physical Review Letters 87 (2001): 137002. 11580617 10.1103/PhysRevLett.87.137002

[83] H. Wu , Y. Wang , and M. N. Ali , “The Global Critical Current Effect of Superconductivity,” 2024.

[84] J. M. Park , S. Sun , K. Watanabe , T. Taniguchi , and P. Jarillo‐Herrero , “Experimental Evidence for Nodal Superconducting Gap in Moiré Graphene,” Science 391, no. 6780 (2025): 79–83. 41196950 10.1126/science.adv8376

[85] E. Navarro‐Moratalla , J. O. Island , S. Mañas‐Valero , et al., “Enhanced Superconductivity in Atomically Thin ${\mathrm{TaS}}_{2}$ ,” Nature Communications 7, no. 1 (2016): 11043. 10.1038/ncomms11043 PMC5512558 26984768

[86] N. Fridman , T. D. Feld , A. Noah , et al., “Anomalous Thickness Dependence of the Vortex Pearl Length in Few‐Layer ${\mathrm{NbSe}}_{2}$ ,” Nature Communications 16, no. 1 (2025): 2696. 10.1038/s41467-025-57817-3 PMC11958829 40164593

[87] R. Córdoba , A. Ibarra , D. Mailly , et al., “3D Superconducting Hollow Nanowires with Tailored Diameters Grown by Focused He+ Beam Direct Writing,” Beilstein Journal of Nanotechnology 11 (2020): 1198–1206. 32832315 10.3762/bjnano.11.104 PMC7431759

[88] A. Nambisan , S. Günzler , D. Rieger , et al., “Quantum Coherent Manipulation and Readout of Superconducting Vortex States,” Nature 653, no. 8113 (2026): 63–67. 42092062 10.1038/s41586-026-10441-7 PMC13149009

[89] W. R. Meier , T. Kong , S. L. Bud'ko , and P. C. Canfield , “Optimization of the Crystal Growth of the Superconductor ${\mathrm{CaKFe}}_{4}{\mathrm{As}}_{4}$ from Solution in the $\text{FeAs-}{\mathrm{CaFe}}_{2}{\mathrm{As}}_{2}-{\mathrm{KFe}}_{2}{\mathrm{As}}_{2}$ ,” Physical Review Materials 1 (2017): 013401.

[90] H. Suderow , I. Guillamón , and S. Vieira , “Compact Very Low Temperature Scanning Tunneling Microscope with Mechanically Driven Horizontal Linear Positioning Stage,” Review of Scientific Instruments 82, no. 3 (2011): 033711. 21456755 10.1063/1.3567008

[91] J. Rodrigo , H. Suderow , S. Vieira , E. Bascones , and F. Guinea , “Superconducting Nanostructures Fabricated with the Scanning Tunnelling Microscope,” Journal of Physics: Condensed Matter 16, no. 34 (2004): R1151.

[92] F. Martín‐Vega , V. Barrena , R. Sánchez‐Barquilla , et al., “Simplified Feedback Control System for Scanning Tunneling Microscopy,” Review of Scientific Instruments 92, no. 10 (2021): 103705. 34717388 10.1063/5.0064511

[93] “LowTemperaturesUAM,” https://github.com/LowTemperaturesUAM, 2025, GitHub.

[94] I. Horcas , R. Fernández , J. M. Gómez‐Rodríguez , et al., “WSXM: A Software for Scanning Probe Microscopy and a Tool for Nanotechnology,” Review of Scientific Instruments 78, no. 1 (2007): 013705. 17503926 10.1063/1.2432410

[95] R. Á. Montoya , S. Delgado , J. Castilla , et al., “Methods to Simplify Cooling of Liquid Helium Cryostats,” HardwareX 5 (2019): e00058.

[96] J. B. Llorens , L. Embon , A. Correa , et al., “Observation of a Gel of Quantum Vortices in a Superconductor at Very Low Magnetic Fields,” Physical Review Research 2 (2020): 013329.

[97] E. Herrera , J. Benito‐Llorens , U. S. Kaluarachchi , et al., “Vortex Creep at Very Low Temperatures in Single Crystals of the Extreme Type‐II Superconductor ${\mathrm{Rh}}_{9}{\mathrm{In}}_{4}S_{4}$ ,” Physical Review B 95 (2017): 134505.

[98] R. Willa , J. A. Galvis , J. Benito‐Llorens , et al., “Thermal Creep Induced by Cooling a Superconducting Vortex Lattice,” Physical Review Research 2 (2020): 013125.

[99] L. Cao , Y. Song , Y.‐B. Liu , et al., “The As‐Surface of an Iron‐Based Superconductor ${\mathrm{CaKFe}}_{4}{\mathrm{As}}_{4}$ ,” Nano Research 14, no. 11 (2021): 3921–3925.

[100] V. S. Stolyarov , I. S. Veshchunov , S. Y. Grebenchuk , et al., “Domain Meissner State and Spontaneous Vortex‐Antivortex Generation in the Ferromagnetic Superconductor ${\mathrm{EuFe}}_{2}$(,” Science Advances 4, no. 7 (2018): eaat1061. 30027117 10.1126/sciadv.aat1061 PMC6044740

[101] Z. Wang , J. O. Rodriguez , L. Jiao , et al., “Evidence for Dispersing 1D Majorana Channels in an Iron‐Based Superconductor,” Science 367, no. 6473 (2020): 104–108. 31896719 10.1126/science.aaw8419

[102] A. Mesaros , G. D. Gu , and F. Massee , “Topologically Trivial Gap‐Filling in Superconducting Fe(Se,Te) by One‐Dimensional Defects,” Nature Communications 15, no. 1 (2024): 3774. 10.1038/s41467-024-48047-0 PMC11074306 38710680

[103] E. Herrera , I. Guillamón , V. Barrena , et al., “Quantum‐Well States at the Surface of a Heavy‐Fermion Superconductor,” Nature 616, no. 7957 (2023): 465–469. 36949204 10.1038/s41586-023-05830-1 PMC10115632

[104] C. Wang , T. He , Q. Han , et al., “Novel Sample‐Thickness‐Dependent Flux Pinning Behaviors of ${\mathrm{KFe}}_{2}{\mathrm{As}}_{2}$ Intercalations in ${\mathrm{CaKFe}}_{4}{\mathrm{As}}_{4}$ Single Crystals,” Superconductor Science and Technology 34, no. 5 (2021): 055001.

[105] G. Springholz , “Strain Contrast in Scanning Tunneling Microscopy Imaging of Subsurface Dislocations in Lattice‐Mismatched Heteroepitaxy,” Applied Surface Science 112 (1997): 12–22.

[106] V. G. Kogan and N. V. Zhelezina , “Field Dependence of the Vortex Core Size,” Physical Review B 71 (2005): 134505. 10.1103/PhysRevLett.95.197001 16384012

[107] A. Fente , E. Herrera , I. Guillamón , et al., “Field Dependence of the Vortex Core Size Probed by Scanning Tunneling Microscopy,” Physical Review B 94 (2016): 014517.

[108] R. Willa , V. B. Geshkenbein , and G. Blatter , “Hessian Characterization of the Pinning Landscape in a Type‐II Superconductor,” Physical Review B 105 (2022): 144504.

[109] M. Buchacek , V. B. Geshkenbein , and G. Blatter , “Role of Rare Events in the Pinning Problem,” Physical Review Research 2 (2020): 043266.

