# Supplementary material for: Direct Observation of Vortex Liquid Droplets in the Iron Pnictide Superconductor CaKFe4As4 at 0.5Tc
Source: Adv Sci (Weinh). 2026 Jul 3:e76387. Online ahead of print. doi: 10.1002/advs.76387 (PMC13334606; doi:10.1002/advs.76387)
Supplement: Supplementary file 1 — Supporting File: advs76387‐sup‐0001‐SuppMat.pdf. [file ADVS-9999-e76387-s001.pdf]

Supplementary Information for: Direct observation of vortex liquid droplets in the iron pnictide superconductor $CaKFe_4As_4$ at $0.5T_c$

Óscar Bou Marqués,[1] Jose A. Moreno,[1] Pablo García Talavera,[1] Mingyu Xu,[2,3,*] Juan Schmidt,[2,3,4] Sergey L. Bud'ko,[2,3] Paul C. Canfield,[2,3] Isabel Guillamón,[1] Edwin Herrera,[1] and Hermann Suderow[1]

[1]*Laboratorio de Bajas Temperaturas, Departamento de Física de la Materia Condensada, Instituto Nicolás Cabrera and Condensed Matter Physics Center (IFIMAC), Unidad Asociada UAM-CSIC, Universidad Autónoma de Madrid, E-28049 Madrid, Spain.*

[2]*Ames National Laboratory, Iowa State University, Ames, Iowa 50011, USA*

[3]*Department of Physics and Astronomy, Iowa State University, Ames, Iowa 50011, USA*

[4]*Departamento de Física, FCEyN, Universidad de Buenos Aires, Buenos Aires 1428, Argentina*

I. SURFACE CHARACTERIZATION AND DEFECTS

In Supplementary Fig. 1, we characterize the surface morphology and defect structure using three representative fields of view. The STM topographies shown in Supplementary Fig. 1(a–c) are plotted using an optimized color scale that highlights atomic-scale height variations not exceeding 0.4 nm. Such small corrugations are characteristic of exposed Ca or K termination layers in $CaKFe_4As_4$ [1, 2].

Height profiles taken along the green arrows in Supplementary Fig. 1(a–c) are presented in Supplementary Fig. 1(d–f) and correspond to linear features marked by red dashed lines in the topographies. Similar linear defects are commonly observed in $CaKFe_4As_4$ and related iron-pnictide superconductors [1, 3–6].

The associated height variations are consistently smaller than an atomic step [7]; however these features exhibit pronounced pair-breaking effects [1]. These linear defects can be associated with intergrown $CaFe_2As_2$ and KFe_2As_2 layers embedded within the $CaKFe_4As_4$ matrix [8–12].

Previous studies have shown that such intergrowths can consist of single atomic layers along the c axis and extend laterally over tens of nanometers in the ab plane [8, 9]. Large strain fields have been observed in the vicinity of these intergrown layers, particularly along the c axis [10, 11, 13]. The resulting surface corrugation caused by an underlying intergrowth is therefore expected to remain below interatomic distances, consistent with the observed sub-atomic height variations. We thus attribute the linear features detected at the surface to the presence of such intergrown layers beneath it.

A correlation exists between the locations of these linear defects and the orientation of the vortex lattice in between them. This is illustrated by comparing the red dashed lines in the topographies (Supplementary Fig. 1(a–c)) with the corresponding zero-bias tunneling conductance maps (Supplementary Fig. 1(g–i)). Vortices are frequently pinned along the linear defects, as exemplified in the bottom-right region of Supplementary Fig. 1(g). The vortex lattice exhibits a preferential orientation aligned with the linear defects. In Supplementary Fig. 2 we present a schematic illustration of a possible arrangement of vortex positions relative to the intergrown $CaFe_2As_2$ and KFe_2As_2 layers.

II. CHARACTERIZATION OF THE SUPERCONDUCTING PROPERTIES

We further characterize the local electronic structure in zero magnetic field and inside vortex cores to demonstrate consistency with previous STM studies. In Supplementary Figure 3(a) we show a zero bias conductance map at 4.2 K and zero magnetic field taken. Three regions spanning several hundred nanometers, separated by linear features similar to those discussed above, are observed. For each region, we calculate the average tunneling conductance, shown in Supplementary Fig. 3(b). The conductance curves are similar across the three regions. The black line corresponds to a fit using the same two-gap-like BCS density of states used in previous work, with the temperature adjusted to 4.2 K[1, 14].

In Supplementary Figure 4(a) we show a conductance map measured at 6 T and 4.2 K. A line scan across a vortex core (Supplementary Figure 4(b)) yields tunneling conductance curves consistent with previous observations [1]. Notably, a peak at zero bias is observed at the vortex core center. This feature, previously reported, arises from Caroli-de Gennes-Matricon states[15]. At higher temperatures, thermal broadening suppresses the visibility of this peak.

* Currently at: Department of Chemistry, Michigan State University, East Lansing, Michigan 48824, USA

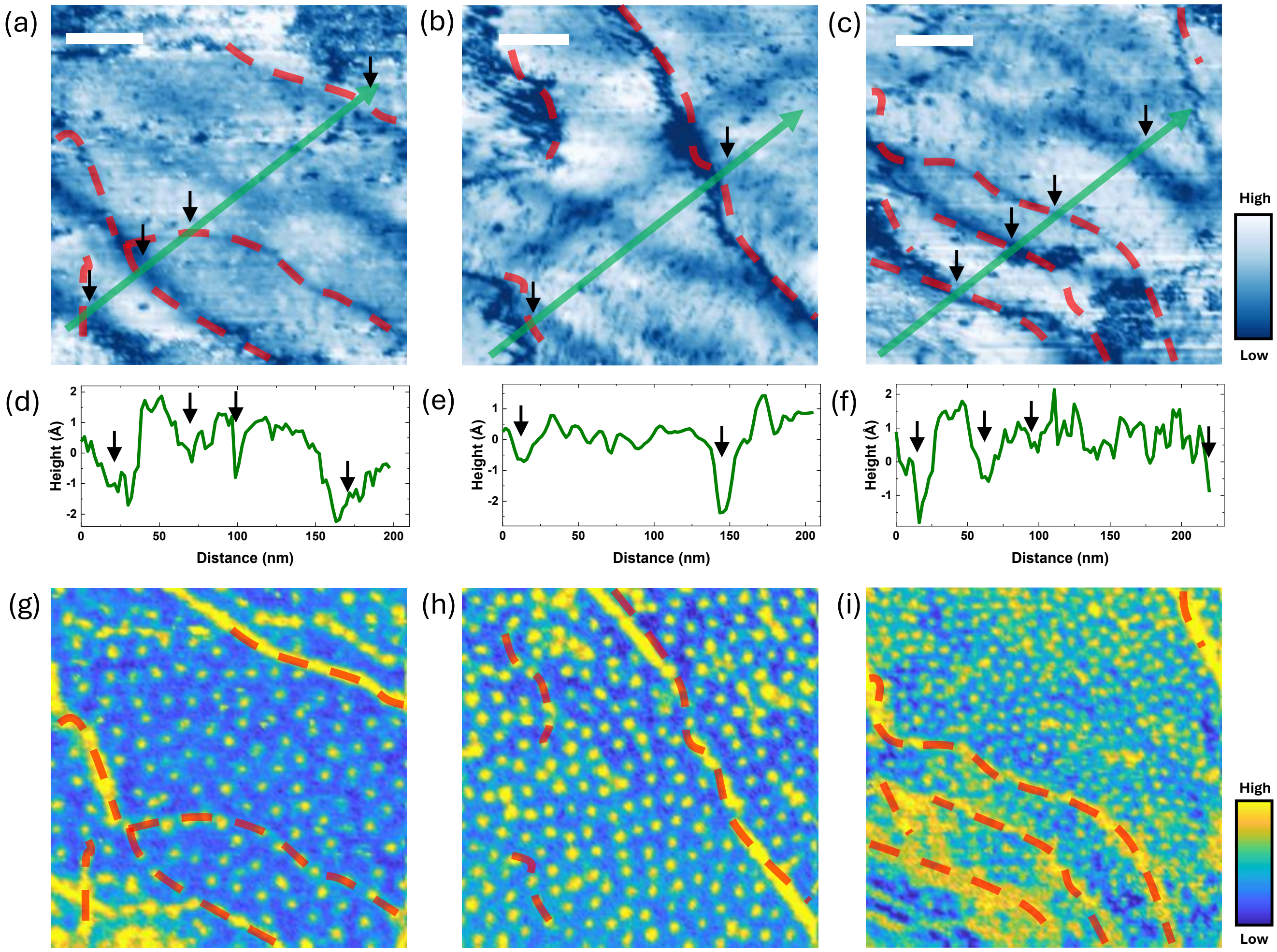

Supplementary Figure 1. **Comparison of topography and zero bias conductance maps**. (a,b,c) STM topography corresponding to the zero bias tunneling conductance map acquired simultaneously and shown in (g-i). White bars correspond to 40 nm. The color scale corresponds to height changes of about 0.4 nm. (d-f) Height profile as a function of position along the line scan shown as a green arrow in (a-c), respectively. Red dashed lines mark the largest defects identified from the STM topography. We mark the position of linear defects with black arrows in both topography and height profiles. (g-i) The zero bias tunneling conductance map acquired simultaneously as topographies in (a-c). The color scale corresponds to conductance changes of about 0.8 times the tunneling conductance normalized to its normal state value (i.e. in many yellow areas, the superconducting gap is very weak but remains generally open). Red dashed lines mark the largest defects identified in the STM topography. Maps taken at 10 K and 6 T for a,g; at 10 K and 10 T for b,h; and at 15 K and 14 T for c,i.

The agreement with earlier reports confirms the robustness of our tunneling spectroscopy measurements. The disappearance of vortex core states at elevated temperatures further underscores the dominant role of thermal smearing in vortex positions obtained by mapping the zero bias conductance.

III. VORTEX CORE SIZE VS TEMPERATURE

In Supplementary Figure 5 we show the dependence of the vortex core size on magnetic field and temperature. At low temperatures, the vortex core size approximately follows the expected zero-temperature field dependence [16]. However, at high temperatures (yellow triangles in Supplementary Figure 5), vortex jitter leads to an effective increase in the vortex core size at large magnetic fields.

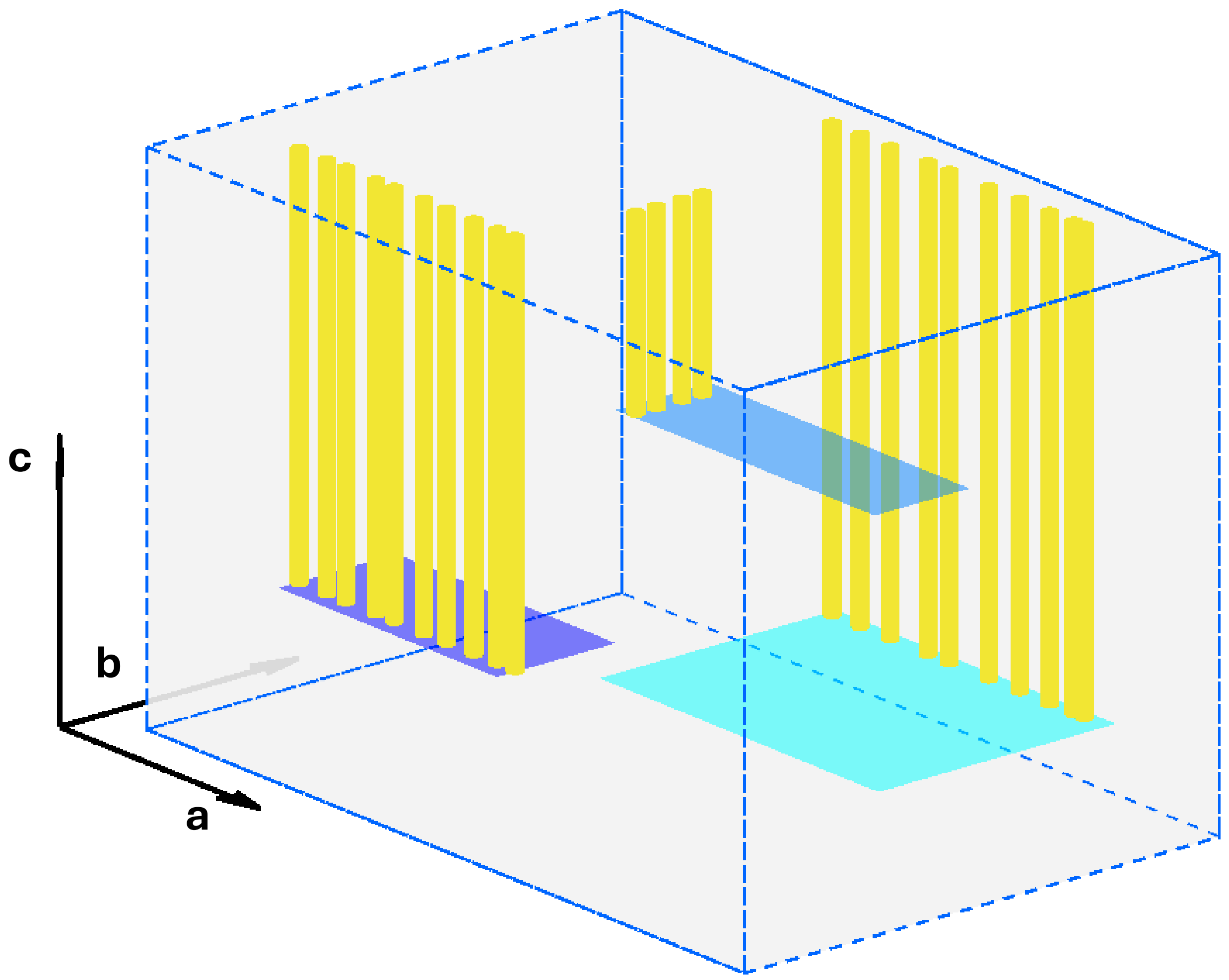

Supplementary Figure 2. **Possible role of defects and intergrowth in vortex pinning of $CaKAs_4Fe_4$.** Schematic representation of the $CaKFe_4As_4$ crystal lattice with an interstitial single layers of $CaFe_2As_2$ or KFe_2As_2 marked with blue planes. We represent the vortices as yellow cylinders that become pinned on the borders of these interstitial layers.

IV. MELTING

Supplementary Fig. 6 shows representative zero-bias tunneling conductance maps acquired at different temperatures (columns) and magnetic fields (rows). Data at 10 K and 4 T and 8 T could not be acquired due to experimental instabilities. At the lowest temperatures, the data display a disordered vortex solid. Upon increasing temperature, spatially localized regions appear—outlined by red contours—in which individual vortices cannot be resolved over distances exceeding several times the average intervortex spacing.

Importantly, these regions coexist within the same field of view with areas where a vortex solid remains clearly visible. At a magnetic field of 8 T, the vortex solid persists up to temperatures very close to the superconducting critical temperature T_c. Only immediately below T_c does the entire field of view transition into a vortex liquid, with individual vortices no longer discernible.

At this field, the melting temperature inferred from the STM measurements closely matches that obtained from macroscopic experiments, indicating that global vortex melting occurs only when local liquid regions have expanded to encompass the entire sample.

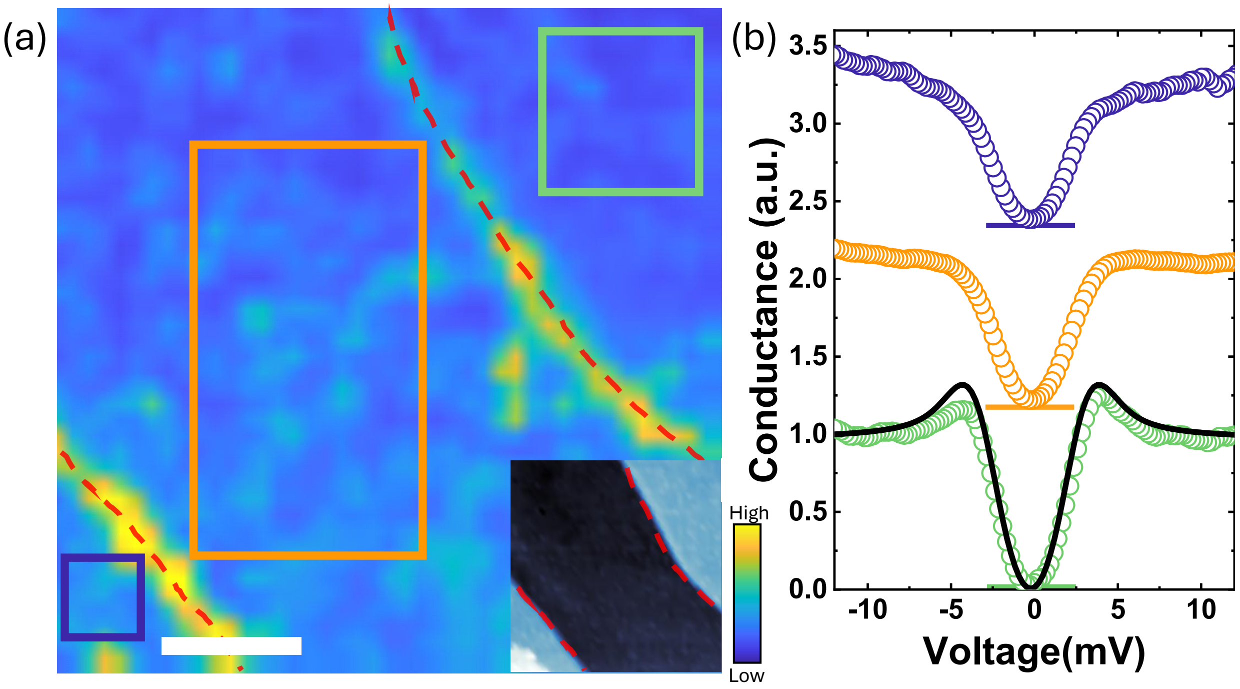

Supplementary Figure 3. **Zero field conductance map at zero bias**. (a) Zero-bias conductance map at zero field (color scale follows the bar in the bottom right, the white bar in the panel is 160 nm long). In the bottom right inset we show the corresponding topography (height difference from black to blue is of 1.6 nm). In the zero bias conductance map we observe three different regions of several hundreds of nanometer size separated by two linear features (yellow lines). The linear features in the tunneling conductance follow the steps in the topography. (b) Tunneling conductance obtained by averaging conductance curves within each of the colored rectangles shown in (a), normalized to its value at high bias voltage. Black line shows the fit we have done using a modified BCS-like form consistent with previous work [1, 14] at a temperature of 4.2 K.

V. VORTEX TRAJECTORIES

Supplementary Figs. 7–12 display representative tunneling conductance maps selected from sequences acquired consecutively within the same field of view. The measurements were performed at the temperatures indicated in each figure and for magnetic fields ranging from 2 T (Supplementary Fig. 7) to 14 T (Supplementary Fig. 12). In each case, we present half of the total number of acquired maps. For every magnetic field, the right-hand column shows the vortex positions identified in all frames as red dots. Red lines connect vortex positions in successive images, illustrating their trajectories over time. Continuity of vortex tracking was ensured by requiring that displacements between consecutive frames remain well below the intervortex spacing at each magnetic field.

For each vortex, the accumulated distance traveled is defined as the sum of its displacements between successive frames. The maximal accumulated distance, d_{max}, corresponding to the most mobile vortex (highlighted in white in Supplementary Figs. 7–12), is plotted in Fig. 4(a) of the main text.

At both low and high magnetic fields (2 T, Supplementary Fig. 7, 4 T, Supplementary Fig. 8, and 14 T, Supplementary Fig. 12), several vortices exhibit displacements that are large compared to the intervortex spacing. In contrast, at intermediate fields (8 T, Supplementary Fig. 10 and 10 T, Supplementary Fig. 11), most vortices remain localized around fixed positions, indicating strongly suppressed mobility.

Notably, d_{max} shows no strong dependence on temperature, consistent with the collapse of the accumulated-distance data onto distinct field-dependent curves in Fig. 4(a) of the main text. This observation reinforces the conclusion that thermally induced vortex motion is primarily governed by the magnetic field and the associated pinning landscape, rather than by temperature alone within the explored range.

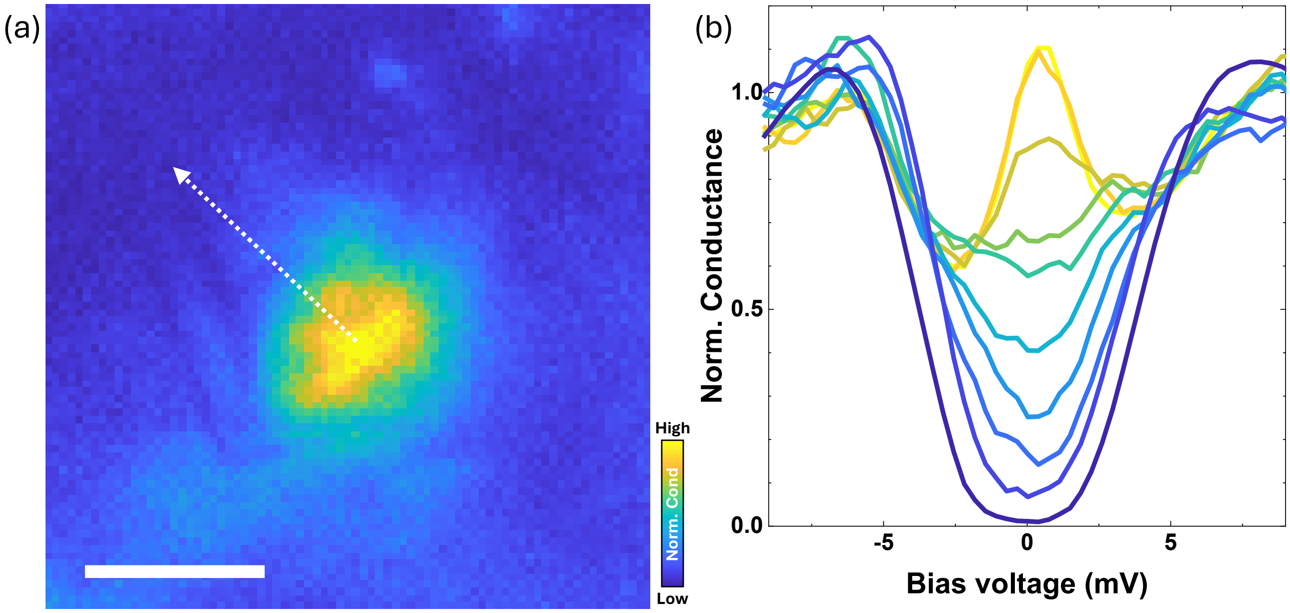

Supplementary Figure 4. **Tunneling conductance in and outside vortex cores**. (a) Zero-bias conductance map taken at 4.2 K and 6 T. White scale bar is 10 nm long. Color scale (bottom right(corresponds to blue for a fully opened gap and a small or negligible zero bias conductance and white for a zero bias conductance equal to the tunneling conductance at high bias. (b) Conductance vs bias voltage along the direction of the white dashed arrow in (a). Color of the curves follow the color scale of (a).

VI. APPARENT VORTEX SPLITTING

Thermally induced changes in vortex positions (Fig. 1(b) of the main text) lead to an enhancement of the zero-bias tunneling conductance around vortex cores compared to the case of static vortices (Fig. 1(a) of the main text). The characteristic timescales of these fluctuations depend on both temperature and the local pinning landscape and typically correspond to rapid vortex motion, as schematically illustrated in Fig. 1 of the main text. However, when vortices are situated between nearby pinning centers, their dynamics may slow down considerably [18, 19]. Below, we discuss an extreme example of such slow vortex dynamics.

Supplementary Fig. 13 shows a zoomed-in view of a selected region measured at 10 T and 10 K. In Supplementary Fig. 13(a,b) (left panels), a feature initially appearing as a single vortex is highlighted with a dashed circle. However, conductance profiles taken across its center (right panels of Supplementary Fig. 13) reveal a clear two-peak structure.

These profiles are well fitted by the sum of two Gaussian functions separated by approximately 10 nm. Given that the average intervortex distance at this field is about 15 nm and the vortex-core radius is below 2 nm (see Supplementary Information Section II), this strongly suggests that the observed feature corresponds to two vortices that are temporally unresolved due to slow fluctuations.

Upon further temporal evolution, this feature evolves into two distinct vortices, as highlighted by the dashed ellipses in the left panels of Supplementary Fig. 13(c–f). The corresponding conductance profiles (right panels of Supplementary Fig. 13(c–f)) now consist of two clearly separated Gaussian peaks. By the end of the sequence (Supplementary Fig. 13(f)), their separation approaches the expected intervortex distance for 10 T.

This slowly fluctuating vortex configuration represents an extreme case of vortex motion occurring on exceptionally long timescales and is observed in a region where the surrounding vortices remain strongly pinned. More generally, the areas where vortex-liquid droplets are identified (red regions in Fig. 2(c–e) of the main text) tend to coincide with locations where subsequent tunneling conductance maps show vortices undergoing small but persistent fluctuations around well-defined mean positions. Representative examples can be seen in the upper left and upper right regions of the conductance map in Fig. 2(b) of the main text.

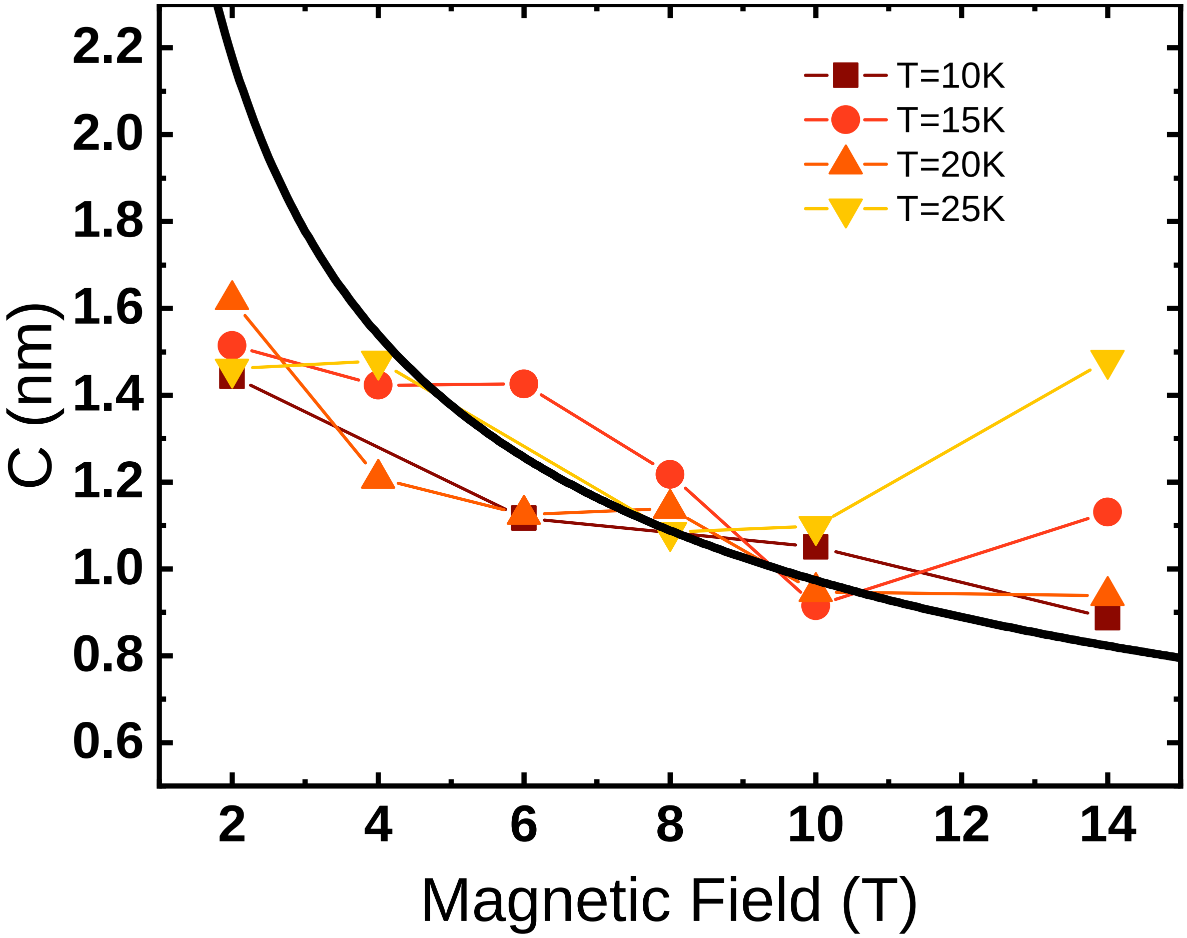

Supplementary Figure 5. **Vortex core size, C as a function of magnetic field**. The continuous black line is given by $C \propto 1/\sqrt{H}$, and is the behavior expected for the zero temperature extrapolation and found at low temperatures in $CaKFe_4As_4$ [16, 17].

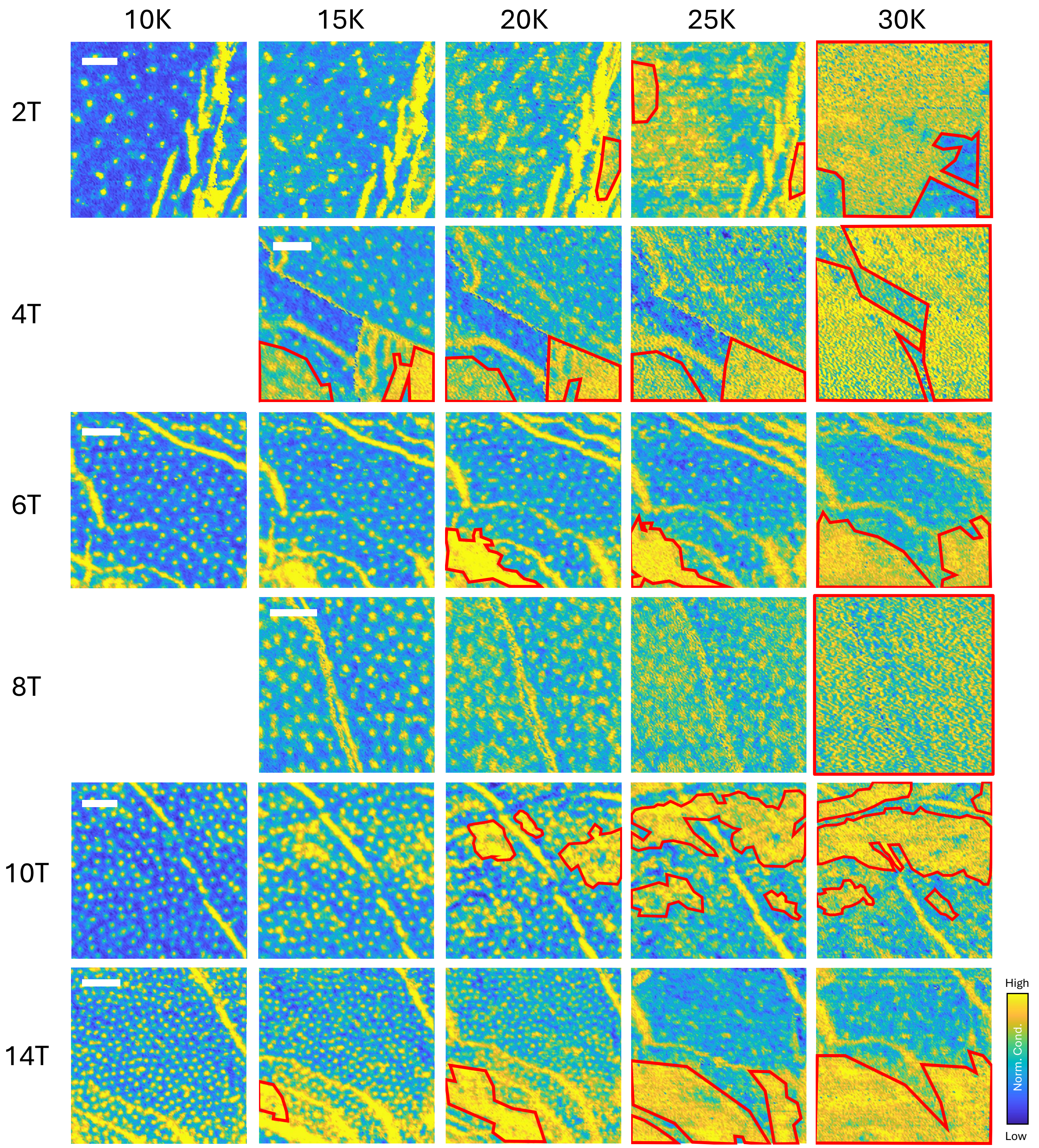

Supplementary Figure 6. **Zero bias tunneling conductance maps as a function of the magnetic field and temperature**. Areas with vortex liquid are marked by red lines. White bars correspond to 40 nm.

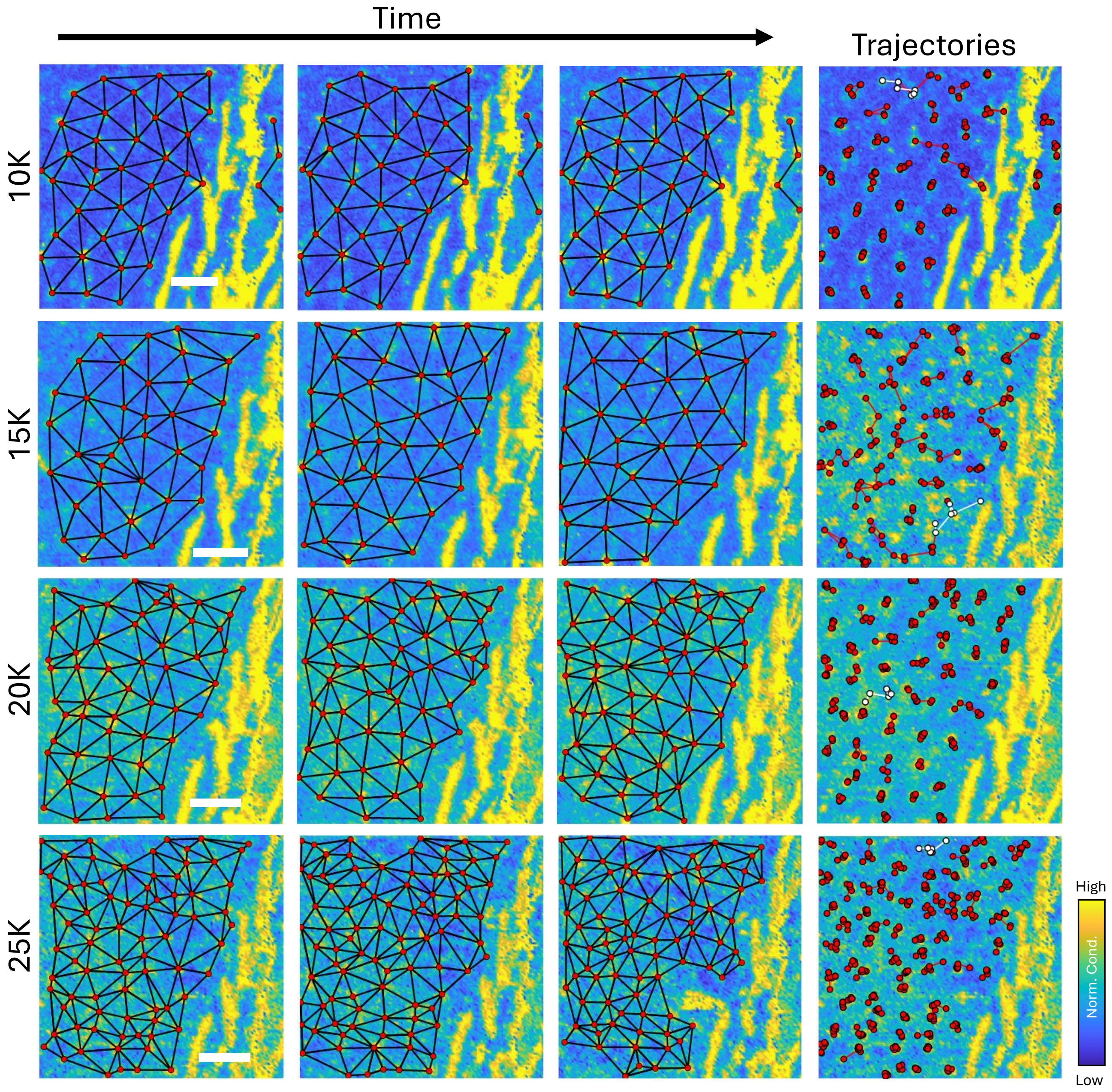

Supplementary Figure 7. **Zero bias tunneling conductance maps at 2 T.** Each row corresponds to images taken at different temperatures (10 K-25 K) in the same field of view. The first three columns show three representative images at the start (left), middle (middle) and end (right) of the sequence. The red dots mark the positions of the vortices, and the black lines connecting them are the triangulation of the vortex positions. The last column shows the extracted trajectories, with red lines joining the vortex positions in different frames. The vortex highlighted in white is the one that shows the largest accumulated distance. White bars correspond to 40 nm.

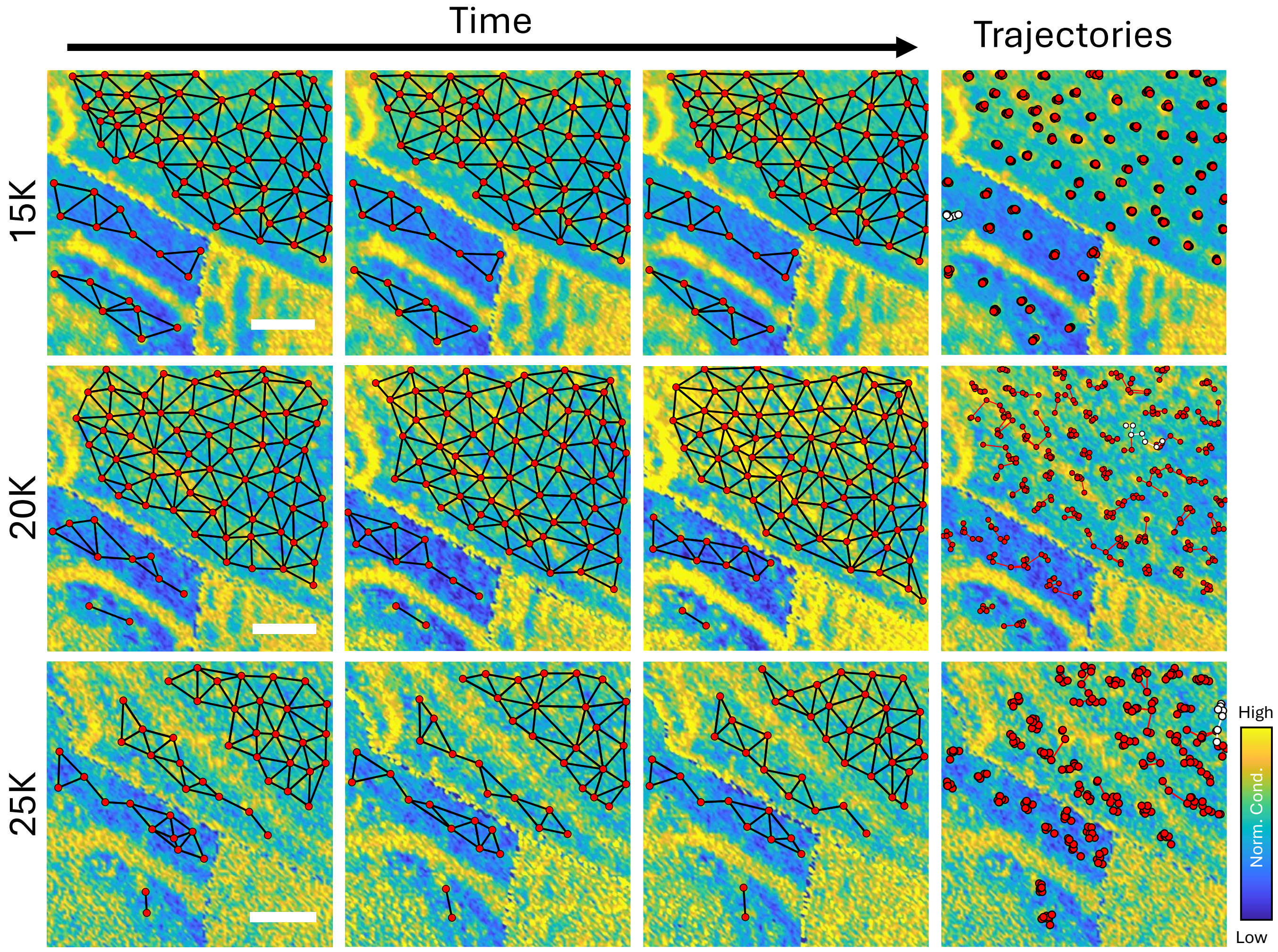

Supplementary Figure 8. **Zero bias tunneling conductance maps at 4 T**. Each row corresponds to images taken at different temperatures (15 K-25 K) in the same field of view. The first three columns show three representative images at the start (left), middle (middle) and end (right) of the sequence. The red dots mark the positions of the vortices, and the black lines connecting them are the triangulation of the vortex positions. The last column shows the extracted trajectories, with red lines joining the vortex positions in different frames. The vortex highlighted in white is the one that shows the largest accumulated distance. White bars correspond to 40 nm.

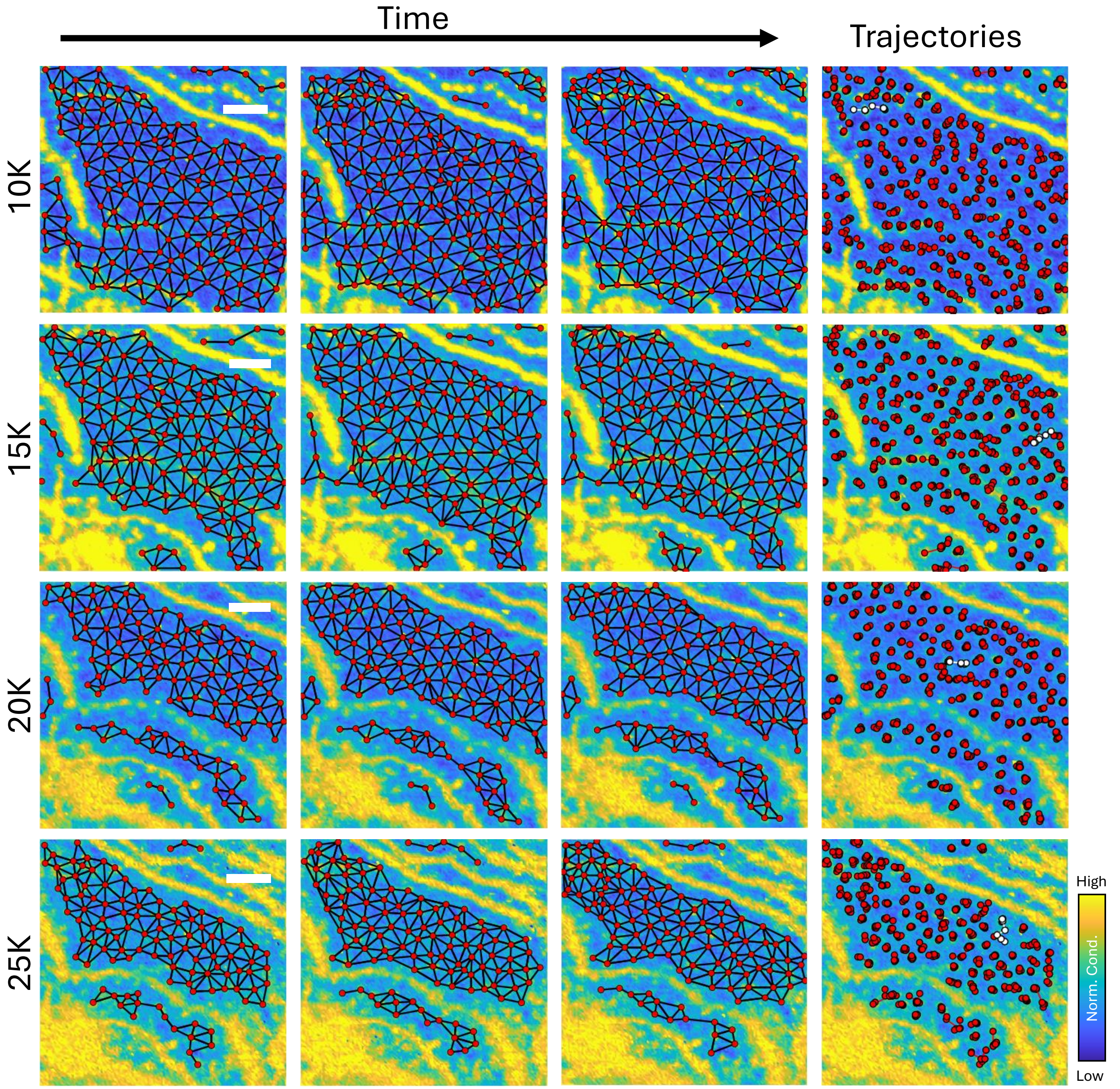

Supplementary Figure 9. **Zero bias tunneling conductance maps at 6 T**. Each row corresponds to images taken at different temperatures (10 K-25 K) in the same field of view. The first three columns show three representative images at the start (left), middle (middle) and end (right) of the sequence. The red dots mark the positions of the vortices, and the black lines connecting them are the triangulation of the vortex positions. The last column shows the extracted trajectories, with red lines joining the vortex positions in different frames. The vortex highlighted in white is the one that shows the largest accumulated distance. White bars correspond to 40 nm.

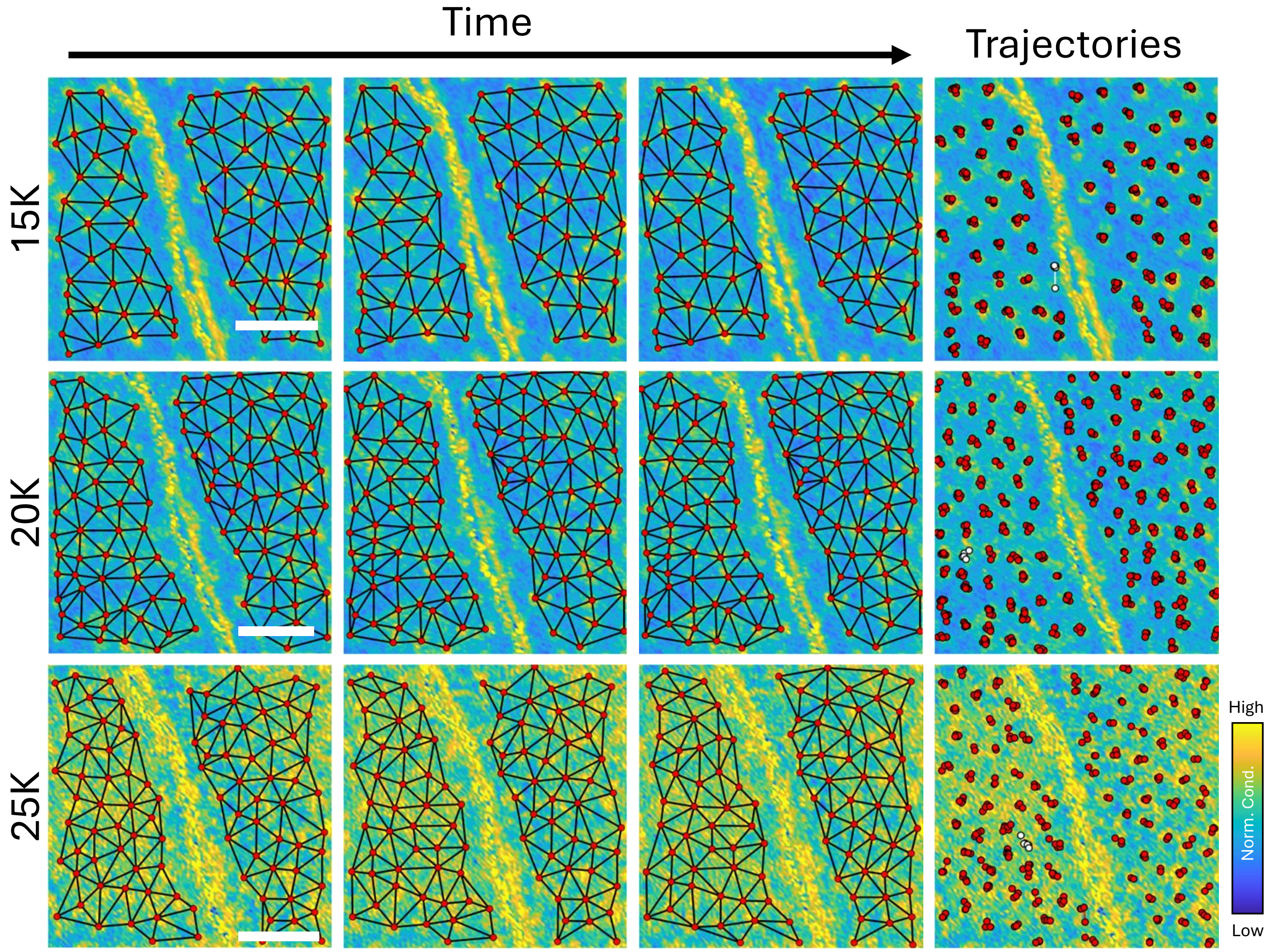

Supplementary Figure 10. **Zero bias tunneling conductance maps at 8 T**. Each row corresponds to images taken at different temperatures (15 K-25 K) in the same field of view. The first three columns show three representative images at the start (left), middle (middle) and end (right) of the sequence. The red dots mark the positions of the vortices, and the black lines connecting them are the triangulation of the vortex positions. The last column shows the extracted trajectories, with red lines joining the vortex positions in different frames. The vortex highlighted in white is the one that shows the largest accumulated distance. White bars correspond to 40 nm.

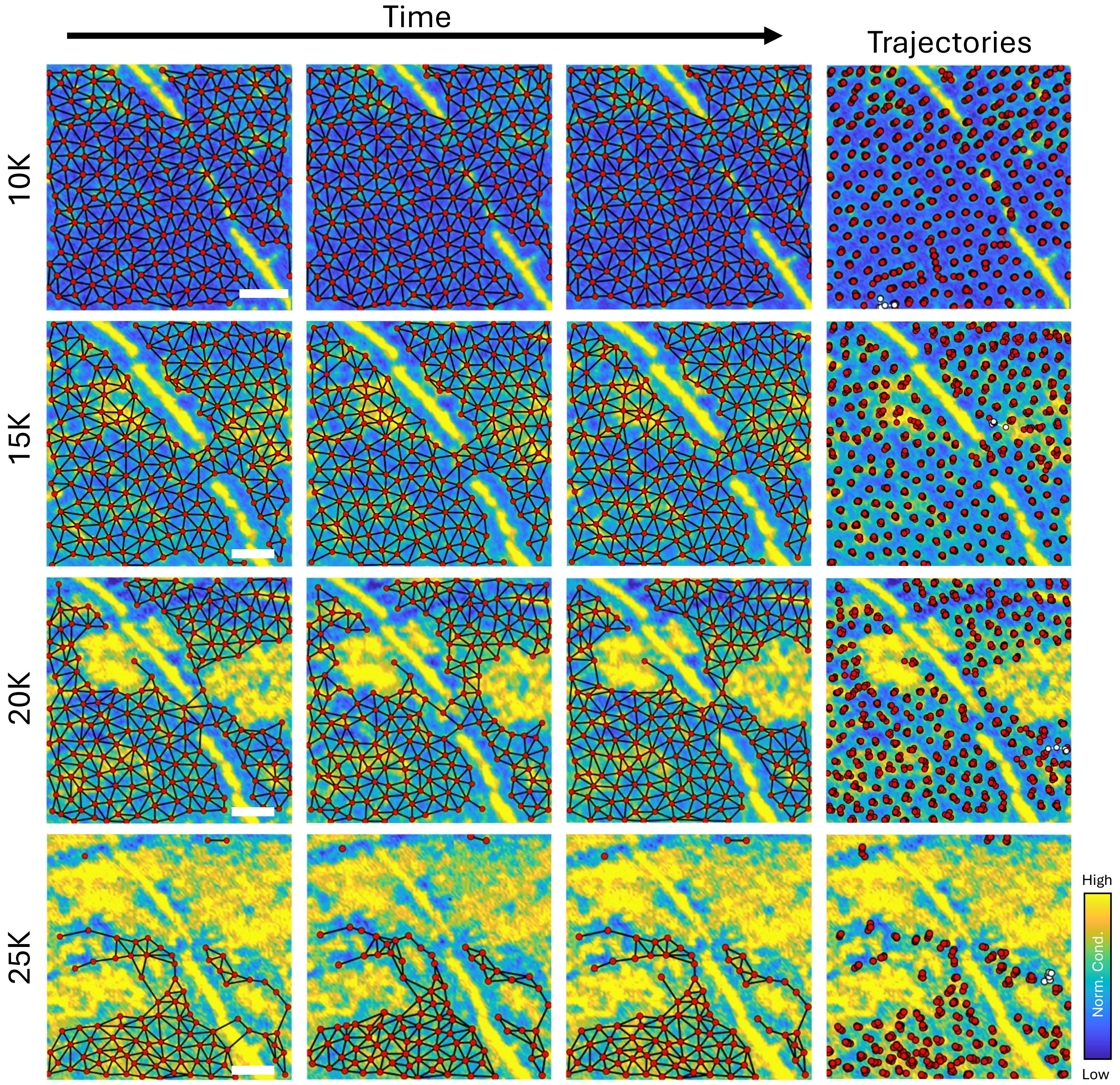

Supplementary Figure 11. **Zero bias tunneling conductance maps at 10 T**. Each row corresponds to images taken at different temperatures (10 K-25 K) in the same field of view. The first three columns show three representative images at the start (left), middle (middle) and end (right) of the sequence. The red dots mark the positions of the vortices, and the black lines connecting them are the triangulation of the vortex positions. The last column shows the extracted trajectories, with red lines joining the vortex positions in different frames. The vortex highlighted in white is the one that shows the largest accumulated distance. White bars correspond to 40 nm.

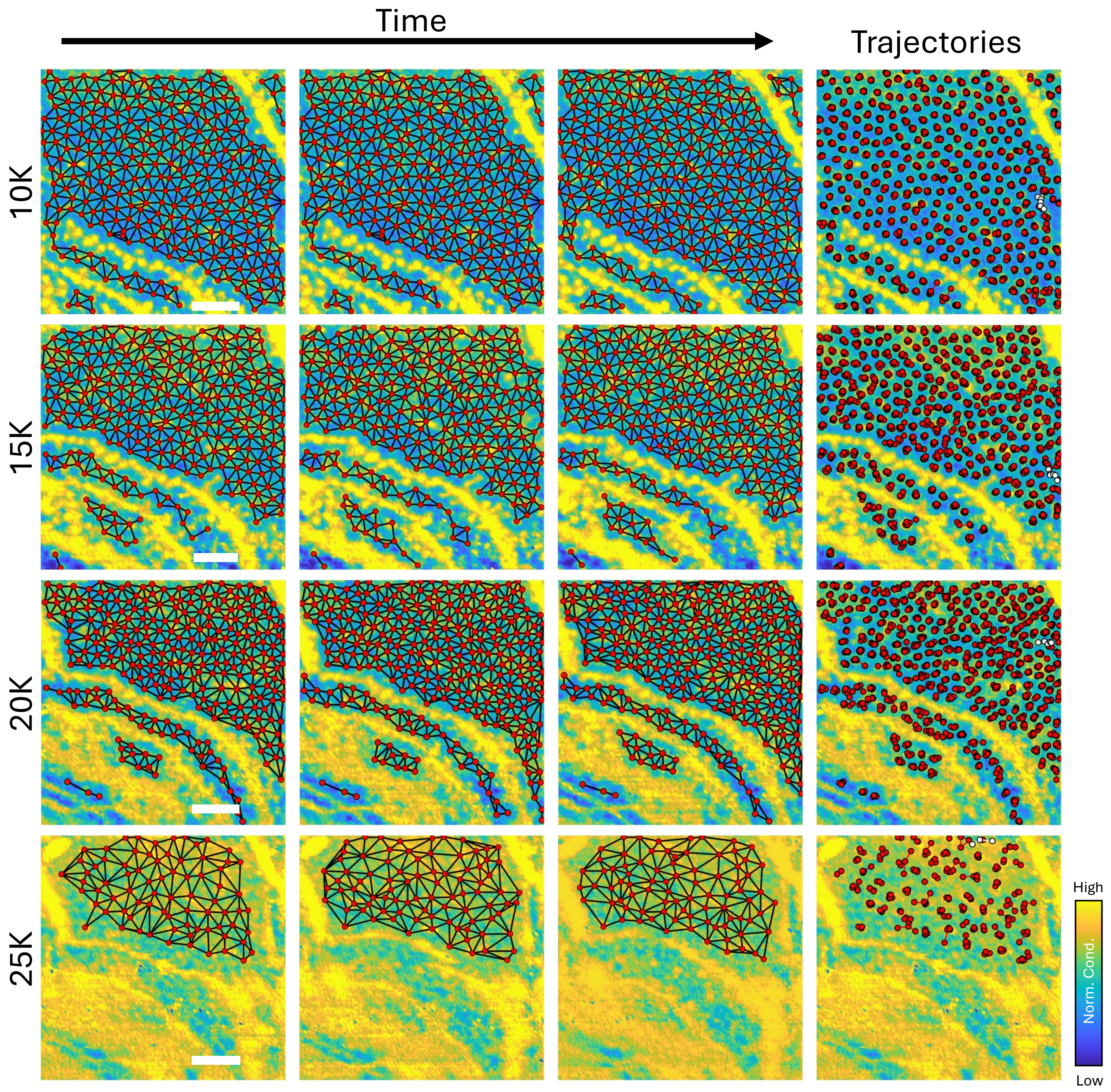

Supplementary Figure 12. **Zero bias tunneling conductance maps at 14 T**.Each row corresponds to images taken at different temperatures (10 K-25 K) in the same field of view. The first three columns show three representative images at the start (left), middle (middle) and end (right) of the sequence. The red dots mark the positions of the vortices, and the black lines connecting them are the triangulation of the vortex positions. The last column shows the extracted trajectories, with red lines joining the vortex positions in different frames. The vortex highlighted in white is the one that shows the largest accumulated distance. White bars correspond to 40 nm.

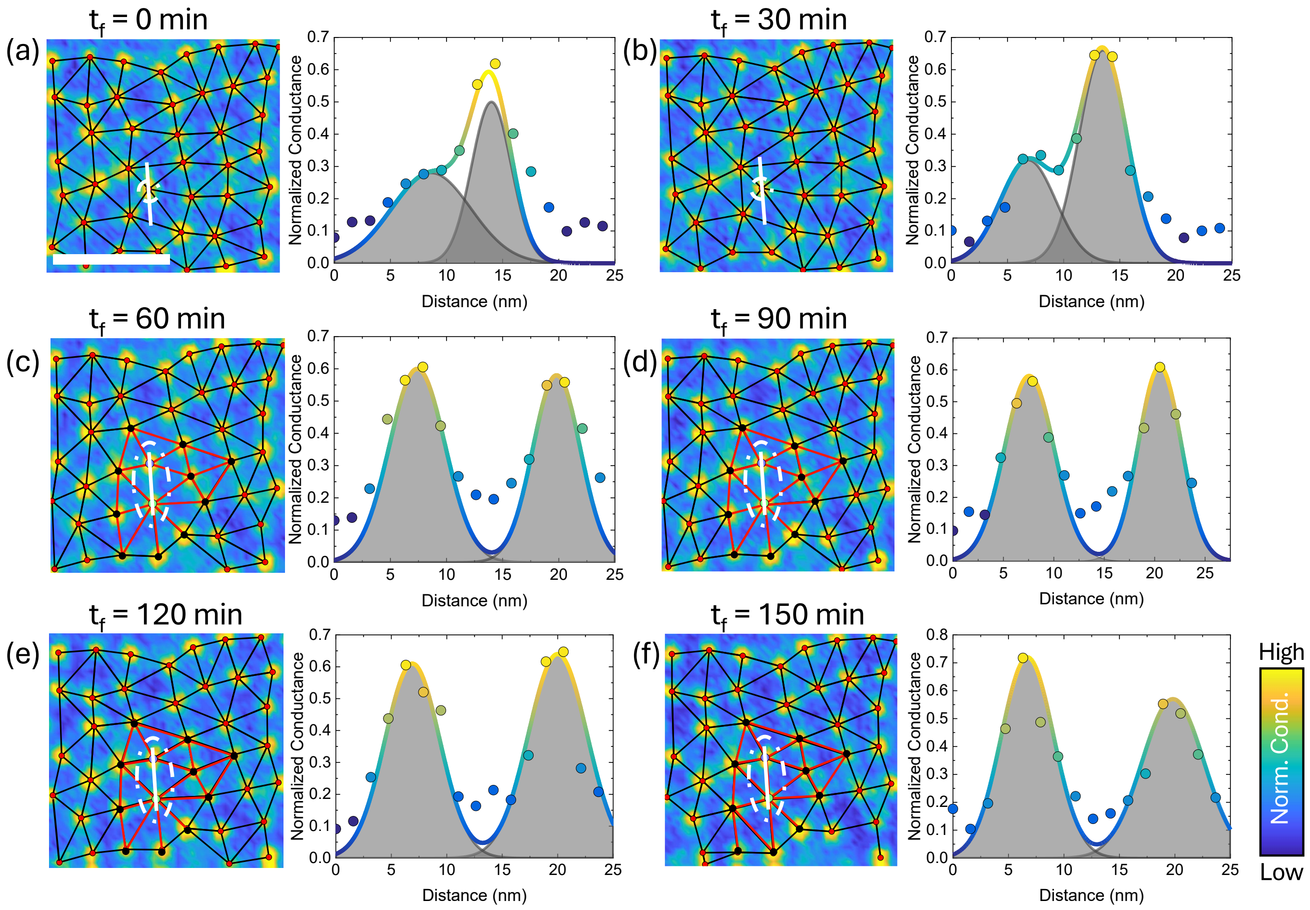

Supplementary Figure 13. **Vortex fluctuations in a small field of view.** In panels (a–f) we focus on the portion of the 10 T maps showing an extreme example of slow vortex creep, presenting subsequently acquired maps from (a) to (f). We show again the zero bias tunneling conductance maps, together with vortex positions (red circles) and nearest neighbor structure (black lines) in the left panels. Red lines connecting the red circles correspond to the lines of the triangulation modified by the splitting of an apparent single vortex in (c). In the right panels, we show profiles of the tunneling conductance as dots and fits to Gaussians as lines. Each Gaussian is shown separately in grey below the fit. We mark with a white dashed circle in the left panels an area which seems to contain a single vortex in (a), which splits into two after 150 minutes in (f). White bar in (a) correspond to 40 nm.

[1] Fente, A. *et al.* Influence of multiband sign-changing superconductivity on vortex cores and vortex pinning in stoichiometric high-T_c $CaKFe_4As_4$. *Phys. Rev. B* **97**, 134501 (2018).
[2] Cao, L. *et al.* The As-surface of an iron-based superconductor $CaKFe_4As_4$. *Nano Research* **14**, 3921–3925 (2021).
[3] Koshelev, A. *et al.* Melting of vortex lattice in the magnetic superconductor $RbEuFe_4As_4$. *Physical Review B* **100**, 094518 (2019).
[4] Stolyarov, V. S. *et al.* Domain Meissner state and spontaneous vortex-antivortex generation in the ferromagnetic superconductor $EuFe_2(As_{0.79}P_{0.21})_2$. *Science Advances* **4**, eaat1061 (2018).
[5] Wang, Z. *et al.* Evidence for dispersing 1D Majorana channels in an iron-based superconductor. *Science* **367**, 104–108 (2020).
[6] Mesaros, A., Gu, G. D. & Massee, F. Topologically trivial gap-filling in superconducting Fe(Se,Te) by one-dimensional defects. *Nature Communications* **15**, 3774 (2024).
[7] Herrera, E. *et al.* Quantum-well states at the surface of a heavy-fermion superconductor. *Nature* **616**, 465–469 (2023).
[8] Ishida, S. *et al.* Unique defect structure and advantageous vortex pinning properties in superconducting $CaKFe_4As_4$. *npj Quantum Materials* **4**, 27 (2019).
[9] Pyon, S. *et al.* Large and significantly anisotropic critical current density induced by planar defects in $CaKFe_4As_4$ single crystals. *Phys. Rev. B* **99**, 104506 (2019).
[10] Sugali, P. K. N. *et al.* Intrinsic defect structures of polycrystalline $CaKFe_4As_4$ superconductors. *Phys. Chem. Chem. Phys.* **23**, 19827–19833 (2021).
[11] Ichinose, A., Pyon, S., Tamegai, T. & Ishida, S. Elucidating the origin of planar defects that enhance critical current density in $CaKFe_4As_4$ single crystals. *Superconductor Science and Technology* **34**, 034003 (2021).
[12] Wang, C. *et al.* Novel sample-thickness-dependent flux pinning behaviors of KFe_2As_2 intercalations in $CaKFe_4As_4$ single crystals. *Superconductor Science and Technology* **34**, 055001 (2021).
[13] Springholz, G. Strain contrast in scanning tunneling microscopy imaging of subsurface dislocations in lattice-mismatched heteroepitaxy. *Applied Surface Science* **112**, 12–22 (1997).
[14] Cho, K. *et al.* Nodeless multiband superconductivity in stoichiometric single-crystalline $CaKFe_4As_4$. *Phys. Rev. B* **95**, 100502 (2017).
[15] Hess, H. F., Robinson, R. B., Dynes, R. C., Valles, J. M. & Waszczak, J. V. Scanning-tunneling-microscope observation of the Abrikosov flux lattice and the density of states near and inside a fluxoid. *Phys. Rev. Lett.* **62**, 214–216 (1989).
[16] Fente, A. *et al.* Field dependence of the vortex core size probed by scanning tunneling microscopy. *Phys. Rev. B* **94**, 014517 (2016).
[17] Kogan, V. G. & Zhelezina, N. V. Field dependence of the vortex core size. *Phys. Rev. B* **71**, 134505 (2005).
[18] Willa, R., Geshkenbein, V. B. & Blatter, G. Hessian characterization of the pinning landscape in a type-II superconductor. *Phys. Rev. B* **105**, 144504 (2022).
[19] Buchacek, M., Geshkenbein, V. B. & Blatter, G. Role of rare events in the pinning problem. *Phys. Rev. Res.* **2**, 043266 (2020).
